# Decline of Yangtze River water and sediment discharge: Impact from natural and anthropogenic changes

**DOI:** 10.1038/srep12581

**Published:** 2015-07-24

**Authors:** S. L. Yang, K. H. Xu, J. D. Milliman, H. F. Yang, C. S. Wu

**Affiliations:** 1 State Key Laboratory of Estuarine and Coastal Research, East China Normal University, Shanghai, 200062, China; 2 Department of Oceanography and Coastal Sciences, Louisiana State University, Baton Rouge, LA 70803, USA; 3 Coastal Studies Institute, Louisiana State University, Baton Rouge, LA 70803, USA; 4 School of Marine Science, Virginia Institute of Marine Science, College of William & Mary, Gloucester Point, VA 23062, USA; 5 Zhejiang Institute of Hydraulics and Estuary, Hangzhou 310020, China

## Abstract

The increasing impact of both climatic change and human activities on global river systems necessitates an increasing need to identify and quantify the various drivers and their impacts on fluvial water and sediment discharge. Here we show that mean Yangtze River water discharge of the first decade after the closing of the Three Gorges Dam (TGD) (2003–2012) was 67 km^3^/yr (7%) lower than that of the previous 50 years (1950–2002), and 126 km^3^/yr less compared to the relatively wet period of pre-TGD decade (1993–2002). Most (60–70%) of the decline can be attributed to decreased precipitation, the remainder resulting from construction of reservoirs, improved water-soil conservation and increased water consumption. Mean sediment flux decreased by 71% between 1950–1968 and the post-TGD decade, about half of which occurred prior to the pre-TGD decade. Approximately 30% of the total decline and 65% of the decline since 2003 can be attributed to the TGD, 5% and 14% of these declines to precipitation change, and the remaining to other dams and soil conservation within the drainage basin. These findings highlight the degree to which changes in riverine water and sediment discharge can be related with multiple environmental and anthropogenic factors.

The magnitude and timing of fluvial discharge of water and sediment have great environmental and social importance, particularly as they are influenced by climatic variability and human activity^1^,^2^. Precipitation, for instance, controls water discharge as well as erosion and sediment transport^2^. Temperature affects both water discharge (via evapotranspiration and snow/glacier melting) and sediment flux (via rock weathering and vegetation-related soil conservation)^3^. Historically, land-use has generated a long-term (millennial scale) increase in sediment discharge in the world’s rivers^4^,^5^,^6^,^7^,^8^, but in recent years constructions of dams and irrigation networks have led to dramatic decreases in sediment discharge from many rivers^9^,^10^,^11^,^12^,^13^. Water storage, withdrawal and consumption, for instance, directly decrease water and sediment discharge. In rivers such as the Ebro^14^, Mississippi^15^, Indus^2^ and Yangtze (Changjiang)^16^, sediment discharge is presently below their pre-human levels, and in some cases, such as the Nile and Colorado rivers, present-day sediment discharge is almost nil^9^.

The decrease in post-dam sediment flux is generally attributed to dam construction only^17^, but water diversion, climate variability and soil conservation can also play significant roles. The dramatic reduction in the sediment flux from the Nile and Colorado Rivers, for example, is partially due to water diversion^17^,^18^. In the Yellow River, 30% of the recent decrease in sediment flux can be explained by decreased precipitation throughout the river’s watershed, 40% by soil conservation practices and 30% by reservoir retention^18^. Similarly, half of the sediment decrease in the Mississippi River can be ascribed to land conservation and levee construction^15^.

The Yangtze River (Fig. 1), the subject of this paper, ranks ninth globally in terms of drainage area (1.8 million km^2^), third in length (6,300 km), fifth in water discharge (900 km^3^/yr), fourth in sediment flux (500 Mt/yr before its decline in the 1970s; Mt: million tons), and first in watershed population (450 million)^2^,^19^,^20^. As such, it can be considered the largest and most important river in Asia. More than 50,000 dams have been constructed throughout the Yangtze’s watershed^16^, and in 2003, the world’s largest hydropower project, the Three Gorges Dam (TGD)^13^, began its operation. Several post-TGD studies have identified other drivers that have affected water and sediment discharge^21^,^22^,^23^,^24^,^25^,^26^,^27^,^28^,^29^,^30^,^31^, but to date there have been few studies that have attempted to quantify relative importance of these drivers in the post-TGD period, which is the subject of this paper.

**Changes in Yangtze Water and Sediment Discharge**. At Datong, the most seaward gauging station, mean annual water discharge between 1950 and 2002 averaged 905 km^3^/yr, and 964 km^3^/yr during the pre-TGD decade (1993–2002). During the post-TGD decade, it declined to a mean of 838 km^3^/yr. In other words, mean annual post-TGD water discharge was 67 km^3^/yr (7.4%) less than the previous 50 years (1950–2002) and 126 km^3^/yr less compared to the relatively wet period of pre-TGD decade (1993–2002), whose discharge was 59 km^3^/yr (6.5%) higher relative to the previous 50 years. Discharges in 2011 and 2006, in fact, were the lowest and 3^rd^ lowest since the 1950s (Fig. 2C5), and the 3^rd^ and 5^th^ lowest since 1865^29^. Although water discharge is clearly a function of precipitation, the post-TGD trend-line was far below the pre-TGD trend-line (Fig. 3A). The difference between these lines reflects the impact of driving factors other than precipitation.

Sediment flux at Datong between 1950 and 1968 averaged 507 Mt/yr, but after 1968, due to dam construction, it declined to an average of 320 Mt/yr during the pre-TGD decade; after closing of the TGD, it declined to 145 Mt/yr. That is, mean annual sediment flux at Datong decreased by 187 Mt/yr between 1950–1968 and 1993–2002 and an additional 175 Mt/yr by 2003–2012. During the drought years of 2006 and 2011 the sediment flux was extremely low (Fig. 2C5). Collectively, more than 95% of the sediment decrease at Datong resulted from the decreased sediment supply from the upper reaches (at Yichang, 40 km downstream from the TGD). Sediment flux is clearly a function of water discharge. The post-TGD trend-line was far below the pre-TGD trend-line (Fig. 3B), suggesting the impact of driving factors other than water discharge.

**Impacts from Various Environmental and Anthropogenic Drivers.** Temporal changes in Yangtze annual water and sediment discharge can be related to both natural and man-made changes. Precipitation and evapotranspiration (as reflected by temperature change) are the most obvious natural changes to the environment (because of the basin’s large size, it is highly doubtful that episodic events, such as floods or earthquakes, would have as much affect as they would on smaller watersheds^2^). The effects of human activities are more varied and can have much greater short-term and lasting impact. Water consumption or land-use change can have significant impact on both water and sediment discharges, but the blocking of sediment transport by dams can have far greater impact. Below we discuss the impacts of these various natural and anthropogenic drivers.

*Impacts from precipitation change.* On average, post-TGD basin-wide precipitation was 3% lower than in the pre-TGD long-term period (1950–2002) and 6% lower than in the pre-TGD decade; precipitation in 2011 was the lowest since the 1950s, and 2006 and 2009 were also very dry years (Fig. 2B5). The pre-TGD decade was a wet period, whereas the post-TGD decade has been a dry period, most reflecting the decadal periodicity of precipitation in the Yangtze Basin^29^.

For the entire Yangtze Basin (upstream of Datong), 69% and 61% of the post-TGD decreased water discharge relative to the pre-TGD periods 1950–2002 and 1993–2002, respectively, can be attributed to the decreased precipitation (Fig. 4A), but decreased precipitation explains only 5% and 14% of the post-TGD decreased sediment flux relative to the pre-TGD periods 1950–1968 and 1993–2002 (Fig. 4B). The relative impact of precipitation on annual water and sediment discharge, however, varied temporally. Precipitation change, for example, explains >90% of the change in water discharge at Datong in the years 2005, 2009, 2010 and 2012, but <20% of the change in 2004 or 2008 water discharge. Similarly, more than 30% of the sediment decrease at Datong in 2011 can be attributed to decreased precipitation, whereas sediment decrease in 2008 and 2010 cannot be explained well by precipitation change (Supplementary Tab. S2–3, online).

The positive relationship between annual water discharge and precipitation is obvious (Fig. 3), since flow is derived primarily from precipitation. Precipitation affects sediment flux mainly through soil erosion and sediment transport. Using data from the Changjiang Water Resource Committee (CWRC), we find that there is a significant positive correlation between soil erosion and water discharge (correlation coefficient *r* = 0.83) in the Yangtze Basin over the post-TGD period. In the upper basin, the river’s main sediment source^32^,^33^, for instance, the precipitation (Fig. 2B1), water discharge (Fig. 2C1), soil erosion and sediment flux (Fig. 2C1) in 2006 and 2011 were all the two lowest seen in the post-TGD period. After the closure of the TGD, downstream channel erosion became the major source of sediment flux to the sea^34^, particularly in 2006 and 2011. In 2006 and 2011, more than 70% of the sediment flux at Datong derived from river erosion. The downstream erosion is positively correlated with precipitation/water discharge. The basin-wide precipitation, water discharge at Datong, and downstream erosion in 2010 and 2012, for instance, were the two largest, whereas the precipitation, discharge and erosion in 2006 and 2011 were the two lowest in the post-TGD decade, based on sediment budget (Supplementary Tab. S1, online). This positive correlation also reflects the impact of reduced precipitation on sediment flux.

*Impacts from temperature change*. Temperature change is a global environmental issue. In river basins, temperature controls evapotranspiration, snow/glacier melt, and vegetation cover, and thereby affects water and sediment discharges. Temperatures within the Yangtze Basin have increased significantly since the mid 1980s in spite of the interannual variability (Fig. 2A). On average, basin-wide temperature increased by 0.4 °C between the 1950s and the pre-TGD decade (1993–2002), and have increased by another 0.4 °C in the post-TGD decade (2003–2012). This temperature increase agrees with the global land temperature increase of the same period^35^. Although it is difficult to quantify this effect in the present study, the covariance between temperature and evapotranspiration is illustrated by the example in 2006, when the basin-wide temperature was the highest (Fig. 2A5.) and the water and sediment discharges were extremely low (Fig. 2C5). The discharge in 2006 was unusually below the trend line between water discharge and precipitation (Fig. 3A), suggesting the important factors other than precipitation that strongly affected the discharge. In 2006, the combined effect of reduced precipitation and TGD can explain only 58% of the water discharge decrease. Water storage in reservoirs excluding the Three Gorges Reservoir (TGR) and water consumption in 2006 were lower than the average over the post-TGD decade (Fig. 2E,F). Assuming that water-soil conservation in 2006 changed the discharge at the same rate as the post-TGD average, more than 10% of the water discharge decrease at Datong in 2006 or more than 2% of the total water discharge decrease in the post-TGD decade relative to the pre-TGD decade can be attributed to higher temperature (Supplementary Tab. S2, online).

In the upper basin of the Yangtze River, the rapid temperature increase over the post-TGD decade (Fig. 2A1) may have accelerated glacial and permafrost thaw. The glaciers in the source area of the Yangtze River totalled 89 km^3^ before the 1980s^36^. Over the past three decades, the area and volume of glaciers have decreased by 18% and 20%, respectively^37^. The rate of ice melt was 0.07 km^3^/yr before 2000^38^ and 0.99 km^3^/yr after 2000^39^. Assuming that the glacier melt increased by 0.9 km^3^/yr from the pre-TGD to post-TGD decades, the increased meltwater would have contributed 9 km^3^ to the water discharge over 2003–2012. This discharge increase amounted to 7% of the discharge from the source area (8% of the Yangtze drainage basin) of the same decade, and resulted in a 0.7% offset of the water discharge decrease at Datong from the pre-TGD to post-TGD decades. We can therefore conclude that the net impact of basin warming on water through evapotranspiration and thawing of glaciers and permafrost has been ca. 1% of the discharge decrease at Datong.

Climate warming may have also affected the sediment flux. Higher temperatures increase the rate of rock weathering^40^. In the source area of the Yangtze, the temperature in September–April is below 0 °C. Basin warming may have shortened the snowfall season and increased the rainfall disturbance of the surface soil. Thawing of glaciers and permafrost may have enlarged the surface erosion area. However, as shown above, higher temperatures may have increased evapotranspiration and resulted in lower water and sediment discharge. Considering the impacts of various aspects, ca.1% of the sediment flux decrease at Datong in the post-TGD decade can be attributed to higher temperature.

*Impacts from water withdrawal/consumption.* Annual water usage/consumption in the Yangtze Basin began being reported in 1997 (Fig. 2F). Mean annual water usage increased by ~20 km^3^/yr, and mean annual water consumption by ~3 km^3^/yr between the pre-TGD and the post-TGD decades, which can explain ca. 2% of the reduced water discharges at Datong (Fig. 4A2). Although an increasing trend in annual water consumption can be qualitatively expected for the period before the pre-TGD decade, considering the rapid increase in population since the 1950s and the rapid increase in economic activity in the Yangtze Basin since the 1980s^20^, it is difficult to quantify its impact on water discharge because of the lack of available data.

There are two types of impacts of water withdrawal on sediment discharge. The first impact is the product of suspended sediment concentration (SSC) and amount of diverted water. Considering that 30% of the water diversion has been from reservoirs with very low SSC (data from CWRC) and that suspended sediments in rivers are mainly distributed near the bottom^41^ where water diversion is not likely conducted, the increased sediment diversion from the pre- to post-TGD decade was estimated to be less than 2 Mt/yr. Because most of these sediments, if had not been diverted, would be trapped in reservoirs, increased sediment withdrawal probably has reduced the sediment flux at Datong by less than 0.5 Mt/yr. The second impact of water withdrawal is the reduction of ability to transport sediment, which is critical to the downstream erosion. The impact of water diversion on the downstream erosion, estimated using the empirical relationships of sediment transport (Equations 16, 17, 18, 19, 20, 21), was less than 1 Mt/yr. Subsequently, <1% of the decreased sediment discharge at Datong from the pre- to post-TGD decades can be attributed to water withdrawal.

*Impacts from water-soil conservation.* Land-use change affects vegetation cover and thereby controls sediment yield^42^. The rate of sediment yield (RSY) in croplands is much higher than that under natural conditions^43^. In the Yangtze Basin, forest cover decreased from 80% about 3000 years BP, to 60% in 1000 years BP, and to 17% in the 1980s^44^. The surface erosion in the Yangtze Basin increased from 364 × 10^3^ km^2^ in the 1950s to 562 × 10^3^ km^2^ in the 1980s^45^. Since then, however, water-soil conservation programs and afforestation have been conducted. The area of water-soil conservation, for instance, increased from ca. 150 × 10^3^ km^2^ in 1993 to 300 × 10^3^ km^2^ in 2012; on average, the cumulative area of water-soil conservation increased by more than 50% from the pre- to post-TGD decades (Fig. 2D). As a result, the vegetation cover throughout the entire Yangtze Basin has increased by 14% in the recent decade^44^. As a result of soil conservation, the area of surface erosion decreased to 520 × 10^3^ km^2^ in the 1990s^46^. Meanwhile, the percentages of very-low-grade and low-grade surface erosion have increased from 38% to 40% and from 34% to 40%, respectively, whereas the percentages of middle-grade, high-grade and very-high-grade surface erosion has decreased from 18% to 15% , from 7% to 3%, and from 3% to 1%, respectively^45^,^46^. The RSYs of the very low-grade, low-grade, middle-grade, high-grade and very-high-grade surface erosion are 1,180, 3,460, 6,330, 10,200 and 14,200 t/km^2^/yr, on average, respectively^45^. Thus, the total RSY decreased from 2.3 Bt/yr (Bt: billion tons) in the mid 1980s to 1.7 Bt/yr at the end of 1990s. The observed RSY was 1.1 Bt/yr in 2002 and 0.88 Bt/yr over 2003–2012^46^. Little information is available for RSY in other years. Assuming that the RSY decreased linearly from ca. 2.3 Bt/y in 1989 to 1.1 Bt/yr in 2002, the average RSY over 1993–2002 would have been ca.1.5 Bt/yr, or ca. 0.6 Bt/yr higher than that in the post-TGD decade.

Using the post-TGD correlation between RSY and water discharge, and employing the water discharge data in the pre-TGD decade, the RSY is predicted to be ca.1.1 Bt/yr. The difference between 1.5 Bt/yr and 1.1 Bt/yr suggests the RSY decrease due to soil conservation. In the Yangtze Basin, the ratio of sediment flux to sediment yield is ca. 0.4^47^. Thus, the soil conservation presumably decreased the sediment discharge by ca. 160 Mt/yr from the pre- to post-TGD decades, before most of the sediment was deposited in cascade reservoirs and lakes. Approximately 80% of the water-soil projects in the Yangtze Basin were conducted upstream of the TGD^42^. Based on the sediment budget, sediment annually trapped in the reservoirs upstream of the TGR is comparable with that in the TGR. Considering the trap efficiency of the TGR (80%), the soil conservation in upstream drainage basin of the Yangtze River would have resulted in a ca. 15 Mt/yr decrease in sediment flux from the TGR towards Datong from the pre- to post-TGD decades. Approximately 20% of the soil conservation projects in the Yangtze Basin were conducted in the middle and lower reaches, particularly in the basin of the Danjiangkou Reservoir in the Hanjiang River. Because ca. 90% of the sediment into the Danjiangkou Reservoir is trapped^33^, as also seen in the Dongting and Poyang Lakes^48^, the soil conservation likely has decreased the sediment flux at Datong by ca. 3 Mt/yr. In conclusion, soil conservation in the Yangtze Basin could explain ca. 10% (18 Mt/yr) of the sediment flux decrease at Datong from the pre- to post-TGD decades. Considering the increasing trend in surface erosion from the 1950s to the 1980s and using the regression relationship between sediment yield and surface erosion^43^, the mean RSY during the period 1950–1968 was estimated to be ca. 1.6 Bt/yr, or 0.1 Bt/yr higher than in the pre-TGD decade and 0.7 Bt/yr higher than in the post-TGD decade. We can then estimate that soil conservation decreased the sediment flux at Datong by ca. 21 Mt/yr, which can explain ca. 6% of the decreased sediment flux at Datong between 1950–1968 and 2003–2012.

Vegetation affects water discharge through transpiration. The afforestation program in the Yangtze Basin have increased vegetation cover and thereby decreased water discharge. Within the Minjiang basin, precipitation increased by 3% but water discharge decreased by 2% from the pre- to post-TGD decades. Based on the close correlation between water discharge and precipitation and considering the water consumption and water impoundment in reservoirs in this sub-basin, a water discharge decrease of ca. 4 km^3^/yr can be attributed to water conservation projects. Using the same method, we estimated the influence of afforestation on water discharge for other sub-basins. For the entire basin at Datong, the total impact of afforestation on the water discharge was a ca. 37 km^3^/yr decrease, which explains approximately 29% of the discharge decrease at Datong from the pre- to post-TGD decades (Fig. 4A2). The impact of afforestation on the water discharge decrease between 1950–2002 and 2003–2012 is difficult to quantify using a water budget because data on water usage/consumption before the 1990s are unavailable. In this case, we estimated that water-soil conservation and increased water consumption together explain ca. 16% of the water discharge decrease (Fig. 4A1). We found that the impact of water-soil conservation on sediment discharge had mainly occurred in the western and northern sub-basins, the main sediment source for the Yangtze River, whereas the impact of water-soil conservation on water discharge had been mainly in the southern sub-basins.

*Impacts from the TGD.* TGD has had two major effects on water discharge. Firstly, continuing water impoundment in the TGR meant that water storage increased from 14 km^3^ in 2003 to 24 km^3^ in 2006, 37 km^3^ in 2008, and 39 km^3^ in 2010. Secondly, increased evaporation in the TGR region due to change from land to water surface (resulted in a water loss of 0.3 km^3^/yr on average). Over the 2003–2012 decade, the combined effects of impoundment and increased evaporation resulted in a downstream water loss of approximately 42 km^3^; this decrease explains ca. 6% and 3% of the water loss at Datong relative to the periods 1950–2002 and 1993–2002, respectively (Fig. 4A). The relative importance of the TGD on water discharge, of course, has varied greatly year to year over the post-TGD decade (Supplementary Tab. S2, online). During the *initial filling* of the TGD, increased water storage and evaporation collectively decreased water discharge by 3 to 14 km^3^/yr. During those years 2004–2005, 2007, 2009 and 2011–2012 when *stable storage* was implemented, only evaporation was important. It should be noted that seasonal water impoundment/release (more than 20 km^3^) has had a much greater effect on shorter-term discharge than the impact on annual discharge^29^,^49^.

Between 2003 and 2012, 80% (182 Mt/yr) of the sediment from upstream was trapped behind the TGD^34^. Although the sediment supply from the basin upstream from the TGR has decreased between the pre- and post-TGD decades^22^,^23^, it was relatively minor compared to sediment retention in the TGR^34^. At Datong, 65% (113 Mt/yr) of the sediment decrease observed between the pre- and post-TGD decades can be attributed to the TGD (Fig. 4B; Supplementary Tab. S3, online). In 2010, more than 95% of the observed decrease in sediment discharge was attributable to the TGD. However, in the drought years of 2006 and 2011, the TGD explained only ca. 30% of the reduced sediment discharge. Compared with the period 1950–1968, the mean sediment flux at Datong over the post-TGD decade decreased by 362 Mt/yr (Supplementary Tab. S3, online), 31% of which was attributable to the TGD impact (Fig. 4B).

*Impacts from other dams.* The number of large reservoirs (each with storage capacity >10^8^ m^3^) within the Yangtze watershed increased from zero in 1950 to ca. 110 in 1992, 140 in 2002, and to 220 in 2012. Correspondingly, the number of mid-sized reservoirs (with storage capacities >10^7^ m^3^) increased from almost zero in 1950 to ~1300 in 2012. The total water storage in large and mid-sized reservoirs increased from nil in 1950 to 40 km^3^ in 1992, to 64 km^3^ in 2002, and 154 km^3^ in 2012 (Fig. 2E). Thus, The water storage in all other large and mid-sized reservoirs increased by 51 km^3^ from the pre- to post-TGD periods. Since this number does not include contribution of the numerous small reservoirs, total water storage probably has increased by ca. 60 km^3^, or 1.5 times greater than that of the TGR. Assuming the increased evaporation is proportional to increased water storage, because of similarity in reservoir bathymetrics and evaporation rate, we attribute ca. 9% and 5% of the post-TGD decreased water discharges at Datong (relative to 1950–2002 and 1993–2002) to water impoundment and increased evaporation in reservoirs other than the TGR (Fig. 4A). Collectively, then, water impoundment and evaporation from all dams along the Yangtze system may account for 8% relative to the pre-TGD decade.

Between periods 1950–1968 and 1993–2002, mean sediment flux at Datong decreased by 187 Mt/yr, more than half of the total decrease coming since 1969 (Supplementary Tab. S1, online). This decrease is mainly ascribed to dam construction, because soil conservation, another major cause of the recent decline in sediment flux in the Yangtze River, did not begin until the end of the 1980s^22^,^23^,^33^,^47^. From the pre- to post-TGD decades, sediment flux from the Wujiang River (Fig. 1B) decreased by 14 Mt/yr, which was mainly attributable to several large reservoirs (with a total storage capacity of ca.13 km^3^). Sediment flux from the Jinshajiang, Minjiang and Jialingjiang Rivers, as gauged at Cuntan Station (Fig. 1B), decreased by nearly 40% between the pre-TGD (330 Mt/yr) and post-TGD decades (190 Mt/yr). Because the main sediment yield areas in these rivers have been the key regions for soil conservation, the decreased sediment flux at Cuntan can be partly attributed to the soil conservation projects. Without detailed data for separating the sediment impacts of the dams and soil conservation, we estimate that approximately half of the sediment decrease from the Jinshajiang River was caused by dams, the other half by soil conservation. A large reservoir (5.8 km^3^ storage capacity) constructed on the major tributary of the Jinshajiang River, for instance, began operation between 1998 and 2000, after which, sediment discharge at the dam site decreased from 27 Mt/yr to ca. 7 Mt/yr after the dam construction. In addition to this dam, many large dams were constructed in the middle and lower Jinshajiang River in the latter half of the post-TGD decade, after as much as or more than 90% of the Jinshajiang sediment may have been trapped in their reservoirs^34^. Because the TGR trapped 80% of the upstream sediment over the 2003–2012 decade^34^, these sediments, if not retained behind dams upstream of the TGD, would mostly have been trapped in the TGR. Said another way, these dams have decreased the sediment outflow from the TGR by ca. 17 Mt/yr. Approximately 10% (2 Mt/yr) of this sediment would have flowed into Lake Dongting. Between the pre- and post-TGD decades, sediment flux from the Hanjiang River was unchanged, and sediment flux into Lakes Dongting and Poyang form their tributaries decreased by 15 Mt/yr, at least partly due to dams. Considering that most sediment into these lakes would be trapped there^45^, dams other than TGD can explain approximately 10% (18 Mt/yr) of the reduced sediment discharge at Datong over the post-TGD decade. This estimation of ca. 10% impact from other dams is in agreement with the difference between the total impact (100%) and the impacts from the TGD (65%), precipitation (14%), soil conservation (10%), water withdrawal etc (1%). Collectively, ca. 57% (205 Mt/yr) of the total decrease in sediment flux at Datong since 1969 (362 Mt/yr) can be attributed to dams other than the TGD (Fig. 4B).

*Other factors.* Other factors include urbanisation, road construction and earthquakes etc. Urbanisation and road construction in China have greatly increased in the most recent decade. When constructing buildings and roads, the soil is exposed to rainfall, and a greater sediment yield can be expected. After the construction, the natural surface is paved with concrete, which decreases water infiltration and increases runoff coefficient. Over the post-TGD period, several violent earthquakes occurred in the Yangtze Basin including the 8.0 magnitude Wenchuan Earthquake that resulted in the deaths of 70 thousand people. The earthquakes generated mudslides and may have increased local sediment yields. However, in view of the basin scale of the Yangtze River, these factors are limited to small regional scales, and their comprehensive impacts on the annual water and sediment discharges are probably very minor compared with the impacts of the aforementioned factors.

## Conclusions

Over the first decade following the construction of the TGD in 2003, the mean annual water discharge from the Yangtze River to the sea was 7% lower than that during the period 1950–2002 (and 13% lower than that during the period 1993–2002); the mean sediment flux decreased by 71% relative to 1950–1968 (before decline) and decreased by 55% compared with 1993–2002. However, these declines in water and sediment discharges were attributable not only to the TGD but also to many other natural and anthropogenic factors. In fact, the TGD can explain only 6% (and 3%) of the above water discharge decrease. Approximately 70% (and 60%) of the water discharge decrease was attributable to precipitation decrease. The post-TGD decade happened to be a dry period and the pre-TGD decade a wet period. Other reservoirs constructed over the post-TGD decade have a combined storage capacity of 1.5 times larger than the TGR and were more important in the water discharge decrease. Water conservation and water consumption were comparable with dams (between 1950–2002 and 2003–2012) or even more important than dams (between the pre- and post-TGD decades) in impacting the water discharge decrease (Fig. 4A). In contrast, the TGD was a dominant cause of the sediment flux decrease. Although thousands of dams other than the TGD can explain 57% of the total decrease in sediment flux since 1969, the TGD alone can explain 31% of this total decrease. In addition, 65% of the pre- to post-TGD decrease in sediment flux can be attributed to the TGD. In comparison, 6% and 5% of the total sediment flux decrease were ascribed to soil conservation and precipitation decline, respectively, and 14%, 10% and 10% of the pre- to post-TGD decreased sediment flux were attributable to precipitation decline, other dams and soil conservation, respectively (Fig. 4B). We can therefore conclude that the decline in river water and sediment discharges observed after construction of a large dam can be generated by multiple natural and anthropogenic factors, and comprehensive evaluation is needed for both knowledge and management.

### Study area

#### The Yangtze River and the TGD

**Figure 1:**
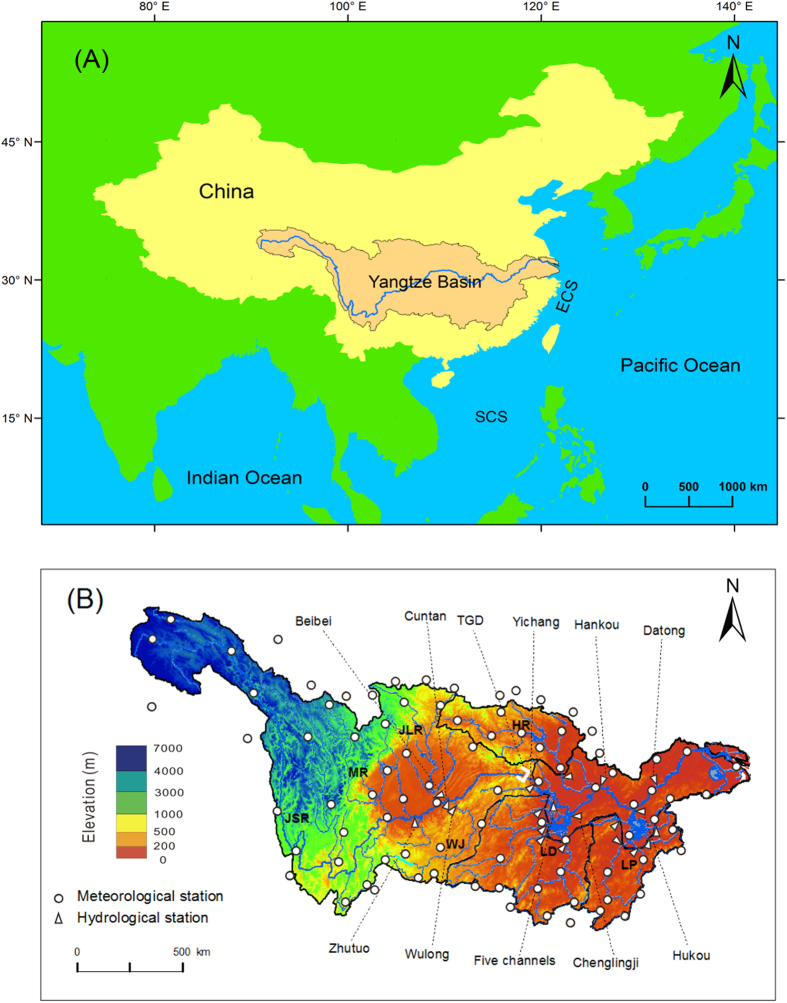
(**A**) Location of the Yangtze River basin on the western coast of the Pacific Ocean showing the East China Sea (ECS) and South China Sea (SCS). (**B**) The Yangtze Basin showing elevation, tributaries, gauging stations and the TGD. JSR: Jinshajiang River; MR: Minjiang River; JLR: Jialingjiang River; HR: Hanjiang River; WR: Wujiang River; LD: Lake Dongting; LP: Lake Poyang. The maps were created using ArcGIS and CorelDRAW Graphics Suite X6.

**Figure 4:**
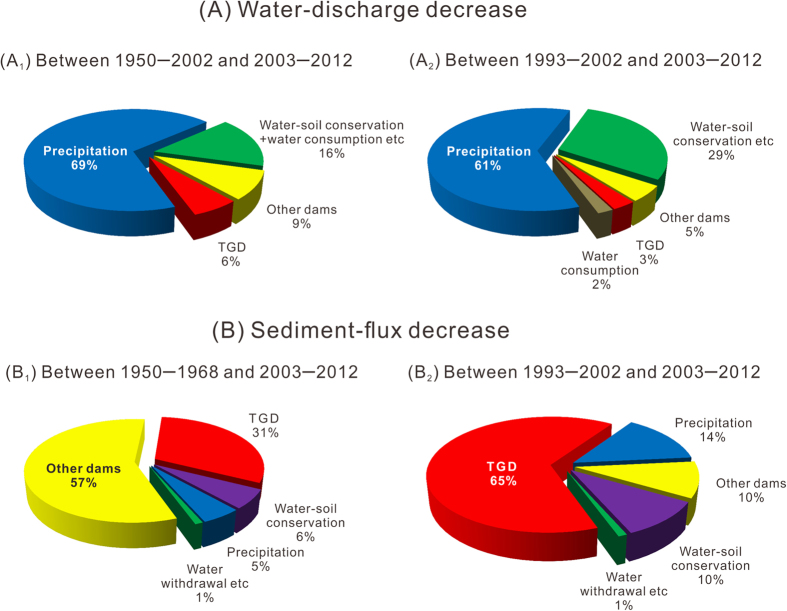
Relative impacts of various factors on (**A**) decreased water discharge, and (**B**) decreased sediment flux at Datong over the post-TGD decade relative to pre-TGD periods.

The Yangtze River originates on the Qinghai-Tibet Plateau at 5100 m above sea level and flows eastward to the East China Sea. The drainage basin is located between 24 °N and 36 °N and is characterized by a subtropical, warm and wet climate (Fig. 1B). The basin-wide precipitation average is ca. 1050 mm/yr, 70%–80% of which is delivered from May to October. About half of the precipitation is lost to evaporation^25^,^50^. The Yangtze Basin is composed of seven sub-basins, the Jinshajiang, Minjiang, Jialingjiang, Hanjiang and Wujiang Rivers, and the tributaries converging at Lakes Dongting and Poyang (Fig. 1B). Precipitation ranges from 730 mm/yr in the Jinshajiang basin (northwestern) to 1560 mm/yr in the Lake Poyang basin (southeastern). In contrast, most sediment is derived from the northern and western sub-basins^25^.

The TGD was constructed at the outlet of the Three Gorges. Yichang gauging station is 40 km downstream from the TGD and 4500 km downstream from the source waters, and receives water and sediment from a basin area of 1,000,600 km^2^ (Fig. 1). Upstream from Yichang, the basin is mainly mountainous, and the river channel is steep and cuts through deep valleys. Downstream from Yichang the terrain consists of flood plains and low hills, and the river becomes wide with a gentle longitudinal slope^51^. About 50% of water and 80% of sediment in the Yangtze originate from the basin upstream of Yichang^33^. The TGR extends more than 600 km under normal operating condition with a surface area of 1100 km^2^. Prior to TGD construction, sediment discharge from upstream was high, and a large amount of sediment was deposited in the middle reaches of the Yangtze. After the closure of the TGD in 2003, significant downstream erosion was observed^34^.

## Methods

### Datasets

Water and sediment data have been collected at twenty-six gauging stations (Fig. 1B) by the Changjiang Water Resource Committee (CWRC)^52^. Suspended sediment discharge is used to represent the total sediment load because the Yangtze River bed load is less than 1% of the suspended load^53^. Data for water impoundment in reservoirs, water usage/consumption, water level and cross-channel topographical profiles were also collected by the CWRC. Monthly air temperature and precipitation data recorded by China Meteorological Administration (CMA) since the 1950s were compiled from 87 gauging stations well distributed within and immediately surrounding the Yangtze Basin^54^ (Fig. 1B). Kriging method^55^ was used as an interpolation method to delineate the spatial distribution of temperature and precipitation and compute basin-wide averages. Data for annual precipitation and sediment discharge missing from the early 1950s (e.g., precipitation in 1950 and sediment discharge at Datong in 1950 and 1952) were reconstructed using regression relationships between precipitation and water discharge and between water and sediment discharges and using uninterrupted water discharge data^56^,^57^.

### Assumptions for differentiating the impacts of major factors on water and sediment discharges

To understand the relative importance of the factors influencing the decline of water and sediment discharges, we need to differentiate and quantify their impacts. This quantification must be based on regression equations. We made assumptions for the establishment of relevant regressions: (1) The dam’s impact on downstream water discharge mainly includes water impoundment and enhanced evaporation in the reservoir; the water impoundment can be calculated using the close regression relationship between water storage and reservoir water level, and the enhanced evaporation can be determined by the increased water surface and the difference in evaporation between the land and the water surface. (2) The dam’s impact on downstream sediment discharge mainly includes sediment trapping in the reservoir and reduction of the ability to transport sediment (due to decreased water discharge). The feedback of downstream sediment transport to dam operation follows hydrological principles and can be simulated using hydrological regression equations. (3) The interannual variability of water discharge in the Yangtze River is mainly affected by precipitation. For a period in which other factors are stable, there will be a close regression relationship between annual precipitation and water discharge for the entire catchment and major sub-basins. This regression equation can be employed to predict precipitation-based annual water discharges for the following periods. The difference between predicted and measured values reflects the impact of other factors. (4) The inherent mechanism by which water discharge affects sediment flux was unchanged from the pre-TGD to post-TGD period, and the pre-TGD hydrological regression is applicable in simulating sediment transport in the middle and lower reaches of the Yangtze River for the post-TGD period. These assumptions simplify the influencing factors and the regression equations lack hydrodynamic processes because it is challenging to simulate the detailed climate-hydrology-landscape interactions. For example, a change in rainfall intensity is sensitive for water discharge, but it cannot be reflected in the regression between annual precipitation and water discharge. Vegetation also changes with precipitation (not only by human activity). This natural change in vegetation also affects the water and sediment discharges. Although simplifications and assumptions must be used in this study, our regression approaches are helpful in examining the relative importance of the driving factors in this study.

### Quantifying the impact of the TGD on water discharge

Water impoundment/release is logically the balance between water inflow and outflow. However, data of water inflow and outflow are often not available. For example, no inflow data are available for the *ungauged areas* surrounding the TGR^34^. Alternatively, we use the relationship between design storage capacity and water level in the TGR to estimate the change in the water storage. The design storage capacity of the TGR at water levels of 135–175 m represents in the following relationship:


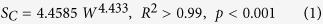


where *S*_*C*_ represents the storage capacity (m^3^) and *W* is the water level (m). Daily records of water levels in the TGR are issued by the CWRC. Using Equation 1, we calculated the change in the water storage for each year between two water levels (on 31 December of the consecutive years). The increases in the water storage in 2003, 2006, 2008 and 2010 due to the rise of the water surface in the TGR reflect the decrease in the annual water discharge at the downstream station. Pan evaporation in the TGR area is ca. 1300 mm/yr^25^. We calculated the pre-TGD average precipitation (960 mm/yr) and runoff (440 mm/yr) in the TGR region. Thus, the evapotranspiration from the original areas that were occupied by the TGR was 520 mm/yr. Based on the cross-channel topographical profiles of the TGR, we calculated the mean width of the water surface (1650 m) at 175 m above sea level, which is in good agreement with the reservoir water surface area (1084 km^2^) and the length of the TGR (663 km) at 175 m above sea level^36^. In the same way, we calculated the mean river width (720 m) and the water surface area (480 km^2^) of the TGR reaches for the pre-TGD period. Prior to water impoundment in the TGR, the mean river water level was 175 m at the uppermost reach of the TGR and ca. 50 m at the TGD site. Based on the data for daily water level in the TGR, we calculated the mean water level and mean water surface area for each year during the period 2003–2012. Using these reservoir water surface areas, we estimated the increased water surface area for each year during the period 2003–2012 (300, 330, 330, 370, 420, 450, 500, 510, 510 and 530 km^2^, respectively). Lastly, we calculated the loss of water due to increased evaporation (1,300 mm/yr −520 mm/yr = 780 mm/yr) of the TGR (0.24, 0.26, 0.26, 0.29, 0.33, 0.35, 0.39, 0.40, 0.40 and 0.41 km^3^/yr from 2003 to 2012, respectively).

### Quantifying the impact of the TGD on sediment discharge

The methodology of Yang *et al.* (2014)^34^ is used to estimate the impact of the TGD on sediment discharge at Yichang and Datong. The impact of the TGD on downstream sediment discharge is the difference between sediment discharge in the non-TGD case and that actually measured over the post-TGD period. As defined by Yang *et al.* (2007a)^58^, the non-TGD case assumes that there was no TGD operation since 2003. Because no new sedimentation was observed before 2003 along the channel where the TGR is now situated^53^, presumably due to high water velocity^51^, it was also assumed that all the sediment deposited in the TGR since 2003 would have been delivered downstream. Thus, estimating sedimentation in the TGR is a prerequisite for predicting downstream sediment discharge in the non-TGD case.

We estimated sedimentation in the TGR using a sediment budget approach; i.e., the difference between sediment inflow and outflow. Sediment inflow was computed mostly at gauging stations upstream from the TGR. Sediment derived from the ungauged area between the upstream gauging stations and the downstream gauging station at Yichang was also estimated, using an empirical water discharge–sediment load model^34^. The sediment budget also took into account riverbed erosion between the TGD and Yichang^34^,^58^. Based on estimated sedimentation in the TGR and the measured downstream water and sediment discharge, sediment delivery along the reach between Yichang and Datong (tidal limit) was predicted using empirical correlations (each with a correlation coefficient of *R*^*2*^ > 0.99^58^.

### Quantifying the impact of precipitation on water discharge

Based on data measured in the pre-TGD decade (1993–2002), correlations were established between annual precipitation and water discharge for different basins:


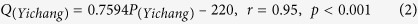



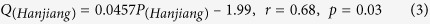



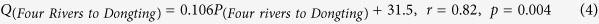



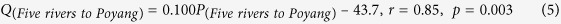



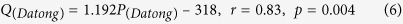


where *Q*_*(Yichang)*_is the water discharge at Yichang, *Q*_*(Hanjiang)*_is the water discharge of the Hanjiang River, *Q*_*(Four Rivers to Dongting)*_ is the sum of water discharge from the four tributaries that join Lake Dongting, *Q*_*(Five rivers to Poyang)*_ is the water discharge from the five tributaries that flow into Lake Poyang and *Q*_*(Datong)*_ is the water discharge at Datong. *P*_*(Yichang)*_, *P*_*(Hanjiang)*_, *P*_*(Four rivers to Dongting)*_, *P*_*(Five rivers to Poyang)*_ and *P*_*(Datong)*_ are the corresponding basin-averaged annual precipitation totals.

A total of three methods were used to predict post-TGD annual water discharge. In Method 1(***Q***_***1***_), precipitation data measured during the post-TGD decade (2003–2012) were used in the relationships described above to predict discharge. The difference between predicted water discharge and the pre-TGD measured water discharge reflects the impact of precipitation change. The difference between the predicted and measured post-TGD water discharge reflects the impact from other driving factors.

In Method 2 (***Q***_***2***_), we predicted the post-TGD water discharge at Datong using Equations 2, 3, 4, 5 and 7, 8, 9:


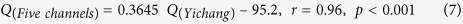



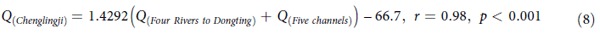



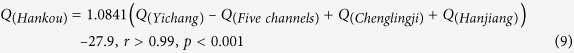



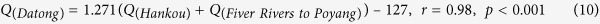


where *Q*_*(Five channels)*_ represents water discharge from the Yangtze River main stem through five channels into Lake Dongting, *Q*_*(Chenglingji )*_ is water discharge at Chenglingji (the confluence of Lake Dongting with the main stem), and *Q*_*(Hankou)*_ is water discharge at Hankou. Water discharge from ungauged areas is reflected in Equations 8, 9, 10.

In Method 3 (***Q***_***3***_), the pre-TGD and post-TGD correlations between precipitation and water discharge were compared in terms of their ability to separate the impact of non-precipitation factors from the impact of precipitation change. The results of these methods are in good agreement and their average is used to quantify the impact of the driving factors.

### Quantifying the impact of precipitation on sediment discharge

Correlations were established between annual sediment discharge (*Q*_*S*_) and water discharge (*Q*) measured in the pre-TGD decade:


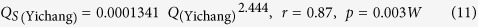



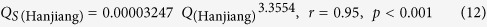



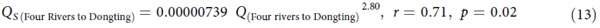



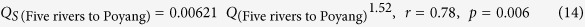



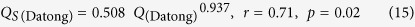


A total of three methods were used. In Method 1 (***S***_***1***_), precipitation-based sediment discharge was predicted using Equations 2, 3, 4, 5, 6 and 11, 12, 13, 14, 15. The difference between the predicted post-TGD sediment discharge and the measured pre-TGD sediment discharge presumably reflects the effect of precipitation change. The difference between the predicted and measured post-TGD sediment discharge reflects the impact from other influencing factors.

Method 2 (***S***_***2***_) was applied at Datong station and considers sediment exchange between the water column and the bed, and between the main river and the lakes. In Method 2 (***S***_***2***_), precipitation-based water and sediment discharge for the four sub-basins was predicted using Equations 2, 3, 4, 5 and 11, 12, 13, 14. Precipitation-based sediment discharge from the main river into Lake Dongting was also predicted following a relationship determined from data measured in the pre-TGD decade:


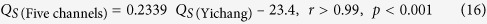


Precipitation-based sediment discharge from Lake Dongting into the Yangtze River at Chenglingji was then predicted using the following equation:


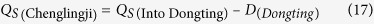


where *Q*_*S* (Into Dongting)_ represents the total sediment discharge into Dongting, and *D*_*(Dongting)*_ is deposition in Dongting:


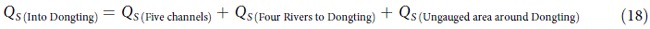



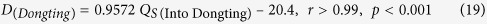


where *Q*_*S* (Ungauged area around Dongting)_ represents sediment supply from the ungauged region around Dongting. This is assumed to be 0.148 *Q*_*S* (Four Rivers to Dongting)_, based on the ratio of the ungauged area to gauged area.

Sediment discharge at Hankou was estimated by:


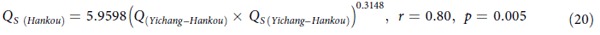


where *Q*_*(Yichang–Hankou)*_and *Q*_*S (Yichang–Hankou)*_ are water and sediment inflows into the river section between Yichang and Hankou based on the mass budget^45^.

Finally, the precipitation-based sediment discharge at Datong Station was estimated from:


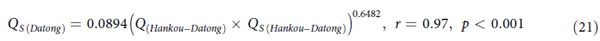


where *Q*_*(Hankou–Datong)*_ and *Q*_*S (Hankou–Datong)*_ represent water and sediment inflows into the section between Hankou and Datong based on the water and sediment budget, taking into account the contributions of Lake Poyang and the ungauged area around the main river.

In Method 3 (***S***_***3***_), we compared the pre- and post-TGD correlations between sediment discharge and water dischargein terms of their ability to separate the impact of non-precipitation factors from the impact of precipitation-governed changes in water discharge.

The accuracy of the above methods depends on the correlation coefficient of the regression equations. A larger correlation coefficient corresponds to a more reliable method. Because the correlation coefficients in this study are generally larger than 0.8, our identified impacts of precipitation and human activities on water and sediment discharges are reasonable.

### Error estimation for regression-based prediction

The error of a regression equation in prediction theoretically derives from the deviation of the data points from the regression trend line. The lower the correlation coefficient, the higher the error of the prediction. Although all the correlation coefficients in this study are high and the correlations are statistically significant (Equations, 2, 3, 4, 5, 6, 7, 8, 9, 10, 11, 12, 13, 14, 15, 19, 20, 21, 22), errors in the prediction need to be evaluated. We used the following standard deviation to show the overall error of a regression-based prediction series:


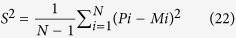


where N is the number of data points, i is the order of the data, P is the predicted value using the regression equation, and M is the measured value. In statistics, Equation 22 can be defined as the residual squared error at the 95% confidence level^56^. For example, in Supplementary Tab. S4 (online), we first established regression equations between annual precipitation and water discharge for odd-year series and even-year series from 1956 to 2002. Then we used these equations to predict the water discharges of the same series. After that, we calculated the difference between the predicted and measured water discharge for each year. Lastly, we calculated the average and standard deviation of the difference for the series. Both of the average differences are zero (Supplementary Tab. S4, online), which is because of the inherent relation of the predicted value to the measured value. The standard deviation is ±43 km^3^/yr for the odd-year series and ±57 km^3^/yr for the even-year series, compared with ca. 900 km^3^/yr for the multi-year average of water discharge (Supplementary Tab. S4, online). To avoid the influence of this inherent relation, we employed the cross-correlation equation of the odd-year series to predict the even-year series, and vice versa. In this case, the averages ± standard deviations of the difference between the two series became −10±57 km^3^/yr and 10 ± 43 km^3^/yr, respectively. That is, the absolute value of the mean difference between predicted and measured values is approximately 1% of the annual water discharge, and the standard deviation remains ca. ±5% of the annual water discharge (Supplementary Tab. S4, online). We can therefore conclude that the influence of the inherent relation of the predicted value to the measured value is very low and can thus be neglected. We used Si to present the error of Pi,


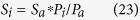


where P_a_ is the average of the individual predicted values. Supplementary Tab. S5 (online) shows an example of the use of Equations 22 and 23. This method has been employed in multiple studies in estimating the error of predicted water and sediment discharges in rivers such as the Yangtze and Pearl Rivers^29^,^56^,^57^. In this study, we also employed it in estimating the error of regression-based predictions. The relative errors of the predicted water discharges based on precipitation-water discharge regression equations are typically <10%, and the relative errors of the predicted sediment fluxes based on precipitation-water discharge-sediment flux regression equations are <20%. The relative errors of the predicted TGD’s impacts on downstream water and sediment discharges are typically <10% (Supplementary Tab. S2–S5, online).

## Additional Information

**How to cite this article**: Yang, S. L. *et al.* Decline of Yangtze River water and sediment discharge: Impact from natural and anthropogenic changes. *Sci. Rep.* **5**, 12581; doi: 10.1038/srep12581 (2015).

## Supplementary Material

Supplementary Information

## Figures and Tables

**Figure 2:**
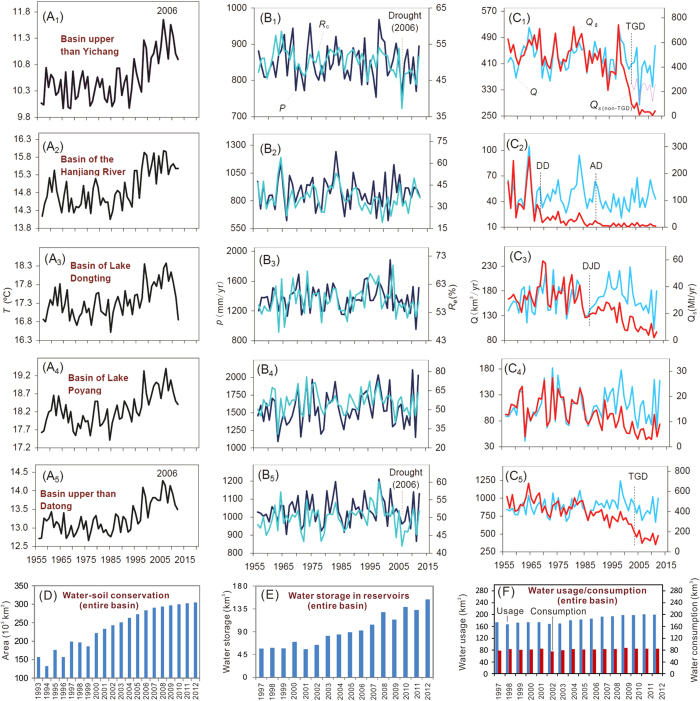
Annual temperature (**A**), precipitation (*P*) and runoff coefficient (*Rc*), defined as basin water to precipitation (**B**), water discharge (*Q*) and sediment flux (*Qs*) (**C**) for the Yangtze Basin and major sub-basins since the 1950s; cumulative area of water-soil conservation (**D**), cumulative water storage in reservoirs (**E**), and annual water usage (**F**) since 1993. *Q*_*S (non-TGD)*_ is sediment discharge predicted for a non-TGD case. DD, AD and DJD represent the Danjiangkou Dam, Ankang Dam and Dongjiang Dam, respectively.

**Figure 3:**
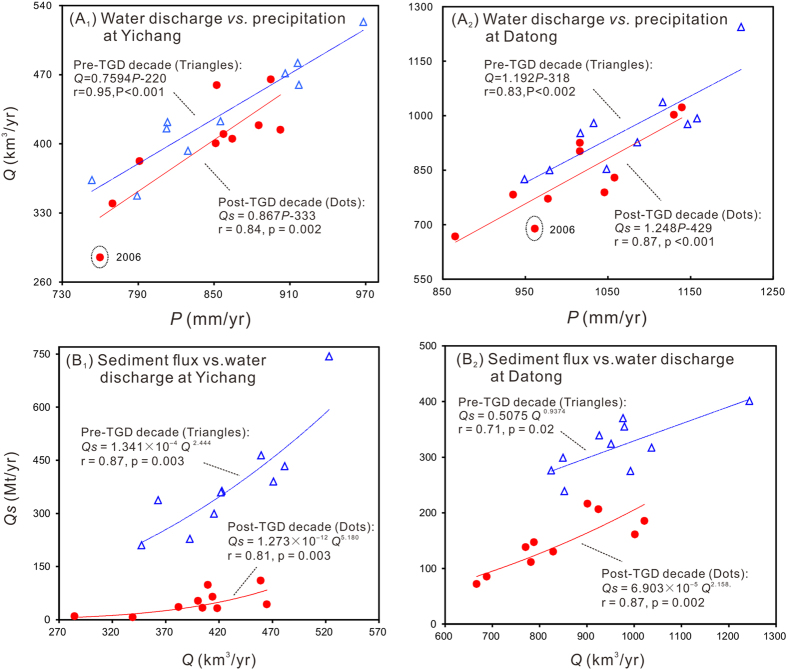
Comparison of precipitation (*P*) ~ water discharge (*Q*) and water discharge (*Q*) ~ sediment flux (*Q*_*s*_) correlations between the pre-TGD (1993–2002) and post-TGD (2003–2012) decades.

## References

[b1] Blum M. D. & Roberts H. H. Drowning of the Mississippi Delta due to insufficient sediment supply and global sea-level rise. Nat. Geosci. 2, 488−491 (2009).

[b2] Milliman J. D. & Farnsworth K. L. River discharge to the coastal ocean: A global synthesis. Cambridge University Press, Cambridge (2011).

[b3] Syvitski J. P. M. & Milliman J. D. Geology, geography, and humans battle for dominance over the delivery of fluvial sediment to the coastal ocean. *J. Geol.* **115**, 1–19.

[b4] Milliman J. D., Qin Y. S., Ren M.-E. & Saito Y. Man’s influence on the erosion and transport of sediment by Asian rivers: the Yellow River (Huanghe) example. J. Geol. 95, 751–762 (1987).

[b5] Saito Y., Yang Z. S. & Hori K. The Huanghe (Yellow River) and Changjiang (Yangtze River) deltas: a review on their characteristics, evolution and sediment discharge during the Holocene. Geomorphology 41, 219–231 (2001).

[b6] Tanabe S. *et al.* Holocene evolution of the Song Hong (Red River) delta system, northern Vietnam. Sedimentary Geol. 187, 29–61 (2006).

[b7] Tamura T. *et al.* Initiation of the Mekong River delta at 8 ka: evidence from the sedimentary succession in the Cambodian lowland. Quaternary Sci. Rev. 28, 327–344 (2009).

[b8] Zong Y. *et al.* An evolutionary model for the Holocene formation of the Pearl River delta, China. The Holocene 19, 129–142 (2009).

[b9] Vörösmarty C. J. *et al.* Anthropogenic sediment retention: major global impact from registered river impoundments. Global Planet. Change 39, 169–190 (2003).

[b10] Syvitski J. P. M., Vörösmarty C. J., Kettner A. J. & Green P. Impact of humans on the flux of terrestrial sediment to the global coastal ocean. Science 308, 376–380 (2005). 15831750 10.1126/science.1109454

[b11] Walling D. E. Human impact on land-ocean sediment transfer by the world’s rivers. Geomorphology 79, 192–216 (2006).

[b12] Wang H., *et al.* Recent changes of sediment flux to the western Pacific Ocean from major rivers in East and Southeast Asia. Earth-Sci. Rev. 108, 80–100 (2011).

[b13] Nilsson C., Reidy C. A., Dynesius M. & Revenga C. Fragmentation and flow regulation of the world’s large river systems. Science 308, 405–408 (2005). 15831757 10.1126/science.1107887

[b14] Guillén J. & Palanques A. A. historical perspective of the morphological evolution in the lower Ebro river. Environmental Geology 30, 174–180 (1997).

[b15] Meade R. H. & Moody J. A. Causes for the decline of suspended-sediment discharge in the Mississippi River system, 1940–2007. Hydrol. Process 24, 35–49 (2010).

[b16] Yang S. L., Milliman J. D., Li P. & Xu K. 50,000 dams later: Erosion of the Yangtze River and its delta. Global Planet. Change 75, 14–20 (2011).

[b17] Wiegel R. L. Nile delta erosion. Science 272, 338–340 (1996). 10.1126/science.272.5260.337a 17735416

[b18] Rodriguez C. A., Flessa K. W. & Dettman D. L. Effects of upstream diversion of Colorado River water on the estuarine bivalve mollusk. Mulinia coloradoensis. Conserv. Biol. 15, 249–258 (2001).

[b19] Zhao C. H., Zhu Z. H. & Zhou D. Z. Worldwide Rivers and Dams. China: Water Conservancy and Hydroelectric Press, , Beijing (2000).

[b20] Yang S. L. *et al.* Trends in annual discharge from the Yangtze River to the sea (1865–2004). Hydrological Sciences-Journal-des Sciences Hydrologiques 50, 825–836 (2005).

[b21] Chen X., Zhang E., Mu H. & Zong Y. A preliminary analysis of human impacts on sediment loads from the Yangtze, China, into the sea. J. Coastal Res. 21, 515–521 (2005).

[b22] Xu K., Milliman J. D., Yang Z. & Wang H. Yangtze sediment decline partly from Three Gorges Dam. EOS 87, 185–196 (2006).

[b23] Yang Z. S. *et al.* Dam impacts on the Changjiang (Yangtze) River sediment load to the sea: the past 55 years and after the Three Gorges Dam. Water Resour. Res. 42, 10.1029/2005WR003970W04407 (2006).

[b24] Zhang Q., Xu C.-Y., Becker S. & Jiang T. Sediment and runoff changes in the Yangtze River basin during past 50 years. J. Hydrol. 331, 511–523 (2006).

[b25] Xu K., Milliman J. D., Yang Z. S. & Xu H. Climatic and anthropogenic impacts on the water and sediment discharge from the Yangtze River (Changjiang), 1950–2005. In: Large Rivers: Geomorphology and Management. Gupta A. (ed.). pp. 609–626. John Wiley & Sons, Chichester (2007).

[b26] Xu J. *et al.* Spatial and temporal variation of runoff in the Yangtze River basin during the past 40 years. Quat. Int. 186, 32–42 (2008).

[b27] Dai Z. *et al.* Runoff characteristics of the Changjiang River during 2006: effect of extreme drought and the impounding of the Three Gorges Dam. Geophys. Res.Lett. 35, 10.1029/2008GL033456 (2008).

[b28] Hu B. Q. *et al.* Sedimentation in the three gorges dam and the future trend of Changjiang (Yangtze River) sediment flux to the sea. Hydrol. Earth Syst. Sci. 13, 2253–2264 (2009).

[b29] Yang S. L. *et al.* Temporal variations in water resources in the Yangtze River (Changjiang) over the Industrial Period, based on reconstruction of missing monthly discharges. Water Resour. Res. 46, W10516, 10.1029/2009WR008589 (2010).

[b30] Gao B., Yang D., Zhao T. & Yang H. Changes in the eco-flow metrics of Upper Yangtze River from 1961 to 2008. J.Hydrol. 448, 30–38 (2012).

[b31] Dai S. B. & Lu X. X. Sediment load change in the Yangtze River (Changjiang): A review. Geomorphology 215, 60–73 (2014).

[b32] Chen Z., Li J., Shen H. & Wang Z. Yangtze River of China: historical analysis of discharge variability and sediment flux. Geomorphology 41, 77–91 (2011).

[b33] Yang S. L. Zhao Q. Y. & Belkin I. M. Temporal variation in the sediment load of the Yangtze River and the influences of the human activities. J. Hydrol. 263, 56–71 (2002).

[b34] Yang S. L. *et al.* Downstream sedimentary and geomorphic impacts of the Three Gorges Dam on the Yangtze River. Earth Sci. Rev. 138, 469–486 (2014).

[b35] Stocker T. F. *et al.* Climate Change 2013: The Physical Science Basis. Cambridge University Press, Cambridge (2013).

[b36] Yao T.-d. & Yao Z.-j. Impacts of glacial retreat on runoff on Tibetan Plateau. Chinese J. Nat. 32, 4–8 (2010).

[b37] Wu J. J. Second investigation on glaciers in China (2014). Available at: <http://news.sciencenet.cn/htmlnews/2014/12/309161.shtm>. Date of access: 20/12/2014.

[b38] Yang J.-P. *et al.* Glacier change and its effect on surface runoff in the source regions of the Yangtze and Yellow rivers. J. Nat. Resour. 18, 595–603 (2003).

[b39] Zhu Y. L., Chen J. & Chen G. C. Changes in water discharge in the source area of the Yangtze River over the past 32 years and influencing factors. J. Yangtze River Sci. Res. Instit. 28, 1–4 (2011).

[b40] Anderson S. P. Glaciers show direct linkage between erosion rate and chemical weathering fluxes. Geomorphology 67, 147–157 (2005)

[b41] Liu J., Yang S. L., Zhu Q. & Zhang J. Dynamics of vertical suspended sediment concentration in the shallow and turbid Yangtze Estuary. Cont. Shelf Res. 90, 96–108 (2014).

[b42] Walling D. Linking land use, erosion and sediment yields in river basins. Hydrobiologia 410, 223–240 (1999).

[b43] Lu X. & Higgitt D. L. Recent changes of sediment yield in the upper Yangtze, China. Environ. Manage. 22, 697–709 (1998). 9680538 10.1007/s002679900140

[b44] Xu H. The analysis for characteristics of vegetation cover change in Yangtze River basin on SPOT VEGETATION data. Master’s Degree Dissertation of Wuhan Agricultural University, Wuhan (2011).

[b45] Yang S. L. *et al.* Effects of human activities on the Yangtze River suspended sediment flux into the estuary in the last century. Hydrol. Earth Syst. Sci. 8, 1210–1216 (2004).

[b46] Ministry of Water Resources of the People’s Republic of China. Water-Soil Conservation Bulletin in China (2003-2012). Available at: <http://www.mwr.gov.cn/zwzc/hygb/zgstbcgb/>. Date of access: 05/01/2015.

[b47] Yang S. L. *et al.* Impact of Dams on Yangtze River Sediment Supply to the Sea and Delta Wetland Response. J. Geophys. Res. 110, F03006, 10.1029/2004JF000271 (2005).

[b48] Yang S. L. *et al.* Effect of deposition and erosion within the main river channel and large lakes on sediment delivery to the estuary of the Yangtze River. J. Geophys. Res. 112, F02005, 10.1029/2006JF000484 (2007b).

[b49] Gao B., Yang D. & Yang H. Impact of the Three Gorges Dam on flow regime in the middle and lower Yangtze River. Quat. Int. 304, 43–50 (2013).

[b50] Xu K. H., Milliman J. D. & Xu H. Temporal trend of precipitation and runoff in major Chinese rivers since 1951. Global Planet. Change 73, 219–232 (2010).

[b51] Chen Z. *et al.* Acoustic Doppler current profiler surveys along the Yangtze River. Geomorphology 85, 155–163 (2007).

[b52] The Changjiang (Yangtze River) Water Resources Committee. Bulletin of Changjiang Sediment (2000–2012). Available at: <http://www.cjw.gov.cn/zwzc/bmgb/nsgb>. Date of access: 15/05/2014.

[b53] Yang S. L. *et al.* Delta response to decline in sediment supply from the Yangtze River: evidence of the recent four decades and expectations for the next half-century. Estuar. Coast. Shelf Sci. 57, 689–699 (2003).

[b54] China Meteorological Administration. Monthly surface climate dataset of China. Available at: <http://cdc.cma.gov.cn/home.do>. Date of access: 15/05/2014.

[b55] Earls J. & Dixon B. Spatial interpolation of rainfall data using ArcGIS: A comparative study. Proceedings of ESRI User Conference 2007, 1–9 (2007). Available at: http://proceedings.esri.com/library/userconf/proc07/papers/papers/pap_1451.pdf. Date of access: 05/01/2015.

[b56] Wang H. *et al.* Reconstruction of sediment flux from the Changjiang (Yangtze River) to the sea since the 1860s, J. Hydrol. 349, 318–332 (2008).

[b57] Wu C. S., Yang S. L., Lei Y. P. Quantifying the anthropogenic and climatic impacts on water discharge and sediment load of the Pearl River (Zhujiang), China (1954-2009), J. Hydrol. 452–453, 190–204 (2012).

[b58] Yang S. L., Zhang J. & Xu X. J. Influence of the Three Gorges Dam on downstream delivery of sediment and its environmental implications, Yangtze River. Geophys. Res. Lett. 34, L10401, 10.1029/2007GL029472 (2007a).

