# Supplementary material for: Decline of Yangtze River water and sediment discharge: Impact from natural and anthropogenic changes
Source: Sci Rep. 2015 Jul 24;5:12581. doi: 10.1038/srep12581 (PMC4648474; doi:10.1038/srep12581)
Supplement: Supplementary Information [file srep12581-s1.pdf]

Decline of Yangtze River water and sediment discharge: Impact from natural and anthropogenic changes

S.L. Yang, K.H. Xu, J.D. Milliman, H. F. Yang, C.S. Wu

Table S1 Post-TGD annual precipitation (*P*), water discharge (*Q*), sediment flux (Q_S), and their changes (%) relative to the averages of the pre-TGD periods

	Upstream basin at Yichang[a] (1006×10^3 km^2)			Hanjiang River basin (142×10^3 km^2)			Lake Dongting basin (209×10^3 km^2)			Lake Poyang basin (122×10^3 km^2)			Basin upper than Datong (tidal limit) (1705×10^3 km^2)		
	P (mm/yr)	*Q* (km^3/yr)	Q_S (Mt/yr)	*P* (mm/yr)	*Q* (km^3/yr)	Q_S (Mt/yr)	*P* (mm/yr)	*Q* (km^3/yr)	Q_S (Mt/yr)	*P* (mm/yr)	*Q* (km^3/yr)	Q_S (Mt/yr)	*P* (mm/yr)	*Q* (km^3/yr)	Q_S (Mt/yr)
Averages over pre-TGD periods															
1950–1968[b]	868	454	541	886	53	119	1385	167	35	1484	104	17	1051	910	507
1950–2002[c]	855	440	491	875	48	54	1397	170	30	1571	112	15	1047	905	432
1993–2002[d]	856	430	378	816	35	7.8	1501	190	19	1730	129	10	1075	964	320
Post-TGD amount (between the brackets are changes in percent relative to the pre-TGD periods 1950–1968 (first row), 1950–2002 (second row), and 1993–2002 (third row), respectively)															
2003	858<br>(–1.2%)<br>(+0.4%)<br>(+0.2%)	410<br>(–9.7%)<br>(–6.8%)<br>(–4.7%)	98<br>(–82%)<br>(–80%)<br>(–74%)	1125<br>(+27%)<br>(+29%)<br>(+38%)	55<br>(+3.8%)<br>(+15%)<br>(+57%)	15<br>(–87%)<br>(–72%)<br>(+92%)	1204<br>(–13%)<br>(–14%)<br>(–20%)	176<br>(+5.4%)<br>(+3.5%)<br>(–7.4%)	18<br>(–49%)<br>(–40%)<br>(–5%)	1300<br>(–12%)<br>(–17%)<br>(–25%)	90<br>(–14%)<br>(–20%)<br>(–30%)	5.3<br>(–69%)<br>(–65%)<br>（–47%）	1016<br>(–3.3%)<br>(–3.0%)<br>(–5.5%)	925<br>(+1.4%)<br>(+2.2%)<br>(–4.0%)	206<br>(–59%)<br>(–52%)<br>(–36%)
2004	903<br>(+4.0%)<br>(+5.6%)<br>(+5.4%)	414<br>(–8.8%)<br>(–5.9%)<br>(–3.7%)	64<br>(–88%)<br>(–87%)<br>(–83%)	876<br>(–1.1%)<br>(+0.1%)<br>(+7.3)	39<br>(–26%)<br>(–19%)<br>(+11%)	7.6<br>(–94%)<br>(–86%)<br>(–2.6%)	1415<br>(+2.2%)<br>(+1.3%)<br>(–5.7%)	150<br>(–10%)<br>(–12%)<br>(–21%)	9.1<br>(–74%)<br>(–70%)<br>(–52%)	1340<br>(–9.7%)<br>(–15%)<br>（–22%）	66<br>(–37%)<br>(–41%)<br>(–49%)	3.1<br>(–82%)<br>(–79%)<br>（–69%）	1046<br>(–0.5%)<br>(–0.1%)<br>(–2.7%)	788<br>(–13%)<br>(–13%)<br>(–18%)	147<br>(–71%)<br>(–66%)<br>(–54%)
2005	852<br>(–1.8%)<br>(–0.4%)<br>(–0.5%)	459<br>(+1.1%)<br>(+4.3%)<br>(+6.8%)	110<br>(–80%)<br>(–78%)<br>(–71%)	1035<br>(+17 %)<br>(+18%)<br>(+27%)	68<br>(+28%)<br>(+42%)<br>(+94%)	17<br>(–86%)<br>(–69%)<br>(+119%)	1303<br>(–6.0%)<br>(–6.7%)<br>(–17%)	153<br>(–8.4%)<br>(–10%)<br>(–19%)	6.4<br>(–82%)<br>(–79%)<br>(–67%)	1570<br>(+5.8%)<br>(–0.1%)<br>(–9.1%)	109<br>(+4.8%)<br>(–2.7%)<br>（–15%）	6.8<br>(–60%)<br>(–55%)<br>（–32%）	1016<br>(–3.3%)<br>(–3.0%)<br>(–5.5%)	902<br>(–0.9%)<br>(–0.3%)<br>(–6.4%)	216<br>(–57%)<br>(–50%)<br>(–31%)
2006	760<br>(–12%)<br>(–11%)<br>(–11%)	285<br>(–37%)<br>(–35%)<br>(–34%)	9.1<br>(–98%)<br>(–98%)<br>(–98%)	762<br>(–14%)<br>(–13%)<br>(-6.7%)	33<br>(–38%)<br>(–31%)<br>(–5.7%)	2.8<br>(–98%)<br>(–95%)<br>(–64%)	1356<br>(–2.1%)<br>(–2.9%)<br>(–9.7%)	155<br>(–7.2%)<br>(–8.8%)<br>(–18%)	10<br>(–71%)<br>(–67%)<br>(–47%)	1630<br>(+9.8%)<br>(+3.8%)<br>（–5.8%）	119<br>(+14%)<br>(+6.3%)<br>(–7.5%)	7.2<br>(–58%)<br>(–52%)<br>（–28%）	962<br>(–8.5%)<br>(–8.1%)<br>(–11%)	689<br>(–24%)<br>(–24%)<br>(–29%)	85<br>(–83%)<br>(–80%)<br>(–73%)
2007	852<br>(–1.8%)<br>(–0.4%)<br>(–0.5%)	400<br>(–12%)<br>(–8.9%)<br>(–6.9%)	53<br>(–90%)<br>(–89%)<br>(–86%)	917<br>(+3.5%)<br>(+4.8%)<br>(+12%)	45<br>(–15%)<br>(–6.3%)<br>(+29%)	8.3<br>(–93%)<br>(–85%)<br>(+6.4%)	1194<br>(–14%)<br>(–15%)<br>(–20%)	140<br>(–16%)<br>(–18%)<br>(–26%)	8.2<br>(–77%)<br>(–73%)<br>(–58%)	1270<br>(–14%)<br>(–19%)<br>(–27%)	75<br>(–28%)<br>(–33%)<br>(–42%)	2.9<br>(–83%)<br>(–81%)<br>（–71%）	978<br>(–6.9%)<br>(–6.6%)<br>(–9.1%)	771<br>(–15%)<br>(–15%)<br>(–10%)	138<br>(–73%)<br>(–68%)<br>(–57%)
2008	886<br>(+2.1%)<br>(+3.6%)<br>(+3.4%)	419<br>(–7.7%)<br>(–4.8%)<br>(–2.7%)	32<br>(–94%)<br>(–93%)<br>(–92%)	916<br>(+3.4%)<br>(+4.7%)<br>(+12%)	33<br>(–38%)<br>(–31%)<br>(–5.7%)	4.6<br>(–96%)<br>(–91%)<br>(–41%)	1417<br>(+2.3%)<br>(+1.4%)<br>(–5.6%)	151<br>(–9.6%)<br>(–11%)<br>(–20%)	7.9<br>(–77%)<br>(–74%)<br>(–59%)	1460<br>(–1.6%)<br>(–7.1%)<br>（–16%）	92<br>(–12%)<br>(–18%)<br>（–29%）	3.9<br>(–77%)<br>(–74%)<br>（–61%）	1058<br>(+0.7%)<br>(+1.1%)<br>(–1.6%)	829<br>(–8.9%)<br>(–8.4%)<br>(–14%)	130<br>(–74%)<br>(–70%)<br>(–59%)
2009	791<br>(–8.9%)<br>(–7.5%)<br>(–7.6%)	382<br>(–16%)<br>(–13%)<br>(–11%)	35<br>(–94%)<br>(–93%)<br>(–91%)	826<br>(–6.8%)<br>(–5.6%)<br>(+1.1%)	45<br>(–15%)<br>(–6.3%)<br>(+29%)	4.9<br>(–96%)<br>(–91%)<br>(–37%)	1167<br>(–16%)<br>(–16%)<br>(–22%)	135<br>(–19%)<br>(–21%)<br>(–29%)	3.7<br>(–89%)<br>(–88%)<br>(–81%)	1320<br>(–11%)<br>(–16%)<br>(–24%)	72<br>(–31%)<br>(–36%)<br>（–44%）	2.9<br>(–83%)<br>(–81%)<br>（–71%）	935<br>(–11%)<br>(–11%)<br>(–13%)	782<br>(–14%)<br>(–14%)<br>(–19%)	111<br>(–78%)<br>(–74%)<br>(–65%)
2010	865<br>(–0.3%)<br>(+1.2%)<br>(+1.0%)	405<br>(–11%)<br>(–8.0%)<br>(–5.9%)	33<br>(–94%)<br>(–93%)<br>(–91%)	923<br>(+4.2%)<br>(+5.5%)<br>(+13%)	66<br>(+25%)<br>(+38%)<br>(+89%)	12<br>(–90%)<br>(–78%)<br>(+54%)	1527<br>(+10%)<br>(+9.3%)<br>(+1.7%)	182<br>(+9.0%)<br>(+7.1%)<br>(–4.3%)	12<br>(–66%)<br>(–60%)<br>(–35%)	2110<br>(+42%)<br>(+34%)<br>（+22%）	159<br>(+53%)<br>(+42%)<br>（+24%）	13<br>(–24%)<br>(–13%)<br>（+30%）	1140<br>(+8.5%)<br>(+8.9%)<br>(+6.0%)	1022<br>(+12%)<br>(+13%)<br>(+6.0%)	185<br>(–64%)<br>(–57%)<br>(–42%)
2011	770<br>(–11%)<br>(–10%)<br>(–10%)	339<br>(–25%)<br>(–23%)<br>(–21%)	6.2<br>(–99%)<br>(–99%)<br>(–98%)	901<br>(+1.7%)<br>(+3.0%)<br>(+10%)	51<br>(–3.8%)<br>(+6.3%)<br>(+46%)	5.4<br>(–95%)<br>(–90%)<br>(–31%)	954<br>(–31%)<br>(–32%)<br>(–36%)	103<br>(–38%)<br>(–39%)<br>(–46%)	2.0<br>(–94%)<br>(–93%)<br>(–90%)	1160<br>(–22%)<br>(–26%)<br>(–33%)	64<br>(–39%)<br>(–43%)<br>（–50%）	4.0<br>(–77%)<br>(–73%)<br>（–60%）	865<br>(–18%)<br>(–17%)<br>(–19%)	667<br>(–27%)<br>(–26%)<br>(–31%)	72<br>(–86%)<br>(–83%)<br>(–78%)
2012	895<br>(+3.1%)<br>(+4.7%)<br>(+4.6%)	465<br>(+2.4%)<br>(+4.7%)<br>(+8.1%)	43<br>(–92%)<br>(–91%)<br>(–89%)	832<br>(–6.1%)<br>(–4.9%)<br>(+2.0%)	43<br>(–19%)<br>(–10%)<br>(+23%)	3.7<br>(–97%)<br>(–93%)<br>(–53%)	1513<br>(+9.2%)<br>(+8.3%)<br>(+0.8%)	180<br>(+7.8%)<br>(+5.9%)<br>(–5.3%)	6.3<br>(–82%)<br>(–79%)<br>(–67%)	2030<br>(+37%)<br>(+29%)<br>(+17%)	158<br>(+52%)<br>(+41%)<br>(+22%)	9.0<br>(–47%)<br>(–40%)<br>（–10%）	1130<br>(+7.5%)<br>(+7.9%)<br>(+5.1%)	1002<br>(+10%)<br>(+10%)<br>(+3.9)	161<br>(–68%)<br>(–63%)<br>(–50%)
Average in 2003–2012	843<br>(–2.9%)<br>(–1.4%)<br>(–1.5%)	398<br>(–12%)<br>(–9.5%)<br>(–7.5%)	48<br>(–91%)<br>(–90%)<br>(–87%)	911<br>(+2.8%)<br>(+4.1%)<br>(+12%)	48<br>(–9.4%)<br>(0%)<br>(+37%)	8.3<br>(–93%)<br>(–85%)<br>(+6.4%)	1305<br>(–5.8%)<br>(–6.6%)<br>(–13%)	152<br>(–9.0%)<br>(–11%)<br>(–20%)	8.4<br>(–76%)<br>(–72%)<br>(–56%)	1520<br>(+2.4%)<br>(–3.2%)<br>(–12%)	100<br>(–3.8%)<br>(–11%)<br>（–22%）	5.8<br>(–66%)<br>(–61%)<br>(–42%）	1015<br>(–3.4%)<br>(–3.1%)<br>(–5.6%)	838<br>(–7.9%)<br>(–7.4%)<br>(–13%)	145<br>(–71%)<br>(–66%)<br>(–55%)

[a]Yichang is 40 km downstream from TGD. [b]Period before decline in sediment flux at Datong due to construction of dams[35]. [c]Long term period prior to construction of the TGD during which precipitation, water and sediment discharges have longest continueous data. [d]Pre-TGD decade which has the same time duration as the post-TGD decade. .

Table S2 Predicted contributions from various driving factors to post-TGD water-discharge changes (relative to pre-TGD averages in 1993–2002 (first row) and 1950–2002 (second row))

	Upstream basin at Yichang (430 km^3/yr in 1993–2002; 440 km^3/yr in 1950–2002) (*Method Q_1*)[a]				Hanjiang River basin (35 km^3/yr in 1993–2002; 48 km^3/yr in 1950–2002) (*Method Q_1*)[a]			Lake Dongting basin (190 km^3/yr in 1993–2002; 170 km^3/yr in 1950–2002) (*Method Q_1*)[a]			Lake Poyang basin (129 km^3/yr in 1993–2002; 110 km^3/yr in 1950–2002) (*Method Q_1*)[a]			Basin upstream from Datong (tidal limit) (964 km^3/yr in 1993–2002; 905 km^3/yr in 1950–2002) (*Method Q_1*; *Method Q_2*)[a]			
	Observed changes (km^3/yr)[b]	Predicted changes (km^3/yr)[c]	Predicted changes (km^3/yr)[d]	Predicted changes (km^3/yr)[e]	Observed changes (km^3/yr)[b]	Predicted changes (km^3/yr)[c]	Predicted changes (km^3/yr)[e]	Observed changes (km^3/yr)[b]	Predicted changes (km^3/yr)[c]	Predicted changes (km^3/yr)[e]	Observed changes (km^3/yr)[b]	Predicted changes (km^3/yr)[c]	Predicted changes (km^3/yr)[e]	Observed changes (km^3/yr)[b]	Predicted changes (km^3/yr)[c]	Predicted changes (km^3/yr)[d]	Predicted changes (km^3/yr)[e]
2003	–20<br>–30	+1 ±0<br>–2 ±0.1	–14.3 ±1	–7.0 ±1<br>–14 ±1	+20<br>+7	+14 ±2<br>+25 ±5	+6 ±2<br>–18 ±5	–15<br>+5	–31 ±2<br>–30 ±2	+16 ±2<br>+35 ±2	–39<br>–22	–45 ±5<br>-32 ±0.6	+12 ±5<br>+10 ±0.6	–39<br>+20	–71 ±5; –101 ±7<br>–44 ±2	–14.3 ±1	+46; +76 ±8<br>+78 ±3
2004	–16<br>–26	+35 ±1<br>+30 ±1	–0.3 ±0.1	–51 ±1<br>–56 ±1	+4<br>–9	+3 ±0.6<br>–1 ±0.2	+1 ±0.6<br>–8 ±0.2	–40<br>–20	–9 ±0.7<br>+2 ±0.2	–31 ±0.7<br>–22 ±0.2	–63<br>–46	–41 ±5<br>–27 ±0.5	–22 ±5<br>–19 ±0.4	–176<br>–117	–35 ±2; –12 ±1<br>–1 ±0.1;	–0.3 ±0.1	–141 ±2;–164 ±1<br>–106 ±0.2
2005	+29<br>+19	–3 ±0.1<br>–6 ±0.2	–0.3 ±0.1	+32 ±0.2<br>+11 ±0.3	+33<br>+20	+10 ±0.2<br>+16 ±3	+23 ±0.2<br>+4 ±3	–37<br>–17	–21 ±2<br>–15 ±1	–16 ±2<br>–2 ±1	–20<br>–3	–16 ±2<br>0	–4 ±2<br>–1 ±0	–62<br>–3	–71 ±5; –56 ±4<br>–44 ±2	–0.3 ±0.1	+9 ±5;–6 ±4<br>+41 ±2;
2006	–145<br>–155	–73 ±3<br>–70 ±3	–9.5 ±1	–62 ±4<br>–75 ±4	–2<br>–15	–2 ±0.4<br>–13 ±3	0 ±0.4<br>–2 ±3	–35<br>–15	–15 ±1<br>–7 ±0.6	–20 ±1<br>–12 ±0.6	–10<br>+7	–11 ±1<br>+6 ±0.1	+1 ±1<br>+1 ±0.1	–275<br>–216	–136 ±10; –166 ±12<br>–122 ±6	–9.5 ±1	–129 ±11;–109 ±13<br>–84 ±7
2007	–30<br>–40	–4 ±0.1<br>–6 ±0.2	–0.3 ±0.1	–26 ±0.2<br>–34 ±0.3	+10<br>–3	+5 ±1<br>+4 ±0.8	+5 ±1<br>–7 ±0.8	– 50<br>–30	–32 ±2<br>–31 ±2	–18 ±2<br>+1 ±2	–54<br>–37	–49 ±6<br>–36 ±0.7	–5 ±6<br>–1 ±0.7	–193<br>–134	–117 ±8; –128 ±9<br>–100 ±5	–0.3 ±0.1	–76 ±8;–65 ±9<br>–34 ±5
2008	–11<br>–21	+22 ±1<br>+18 ±1	–14.1 ±1	–19 ±2<br>–25 ±2	–2<br>–15	+5 ±1<br>+3 ±0.6	–7 ±1<br>–18 ±0.6	–39<br>–19	–9 ±0.7<br>+3 ±0.2	–30 ±0.7<br>–22 ±0.2	–37<br>–20	–29 ±3<br>–13 ±0.3	–8 ±3<br>–7 ±0.3	–135<br>–76	–21 ±1;–15 ±1<br>+16 ±0.8	–14.1 ±1	–100 ±2;–106 ±2<br>–78 ±2
2009	–58<br>–48	–49 ±2<br>–49 ±2	–0.4 ±0.1	–9 ±2<br>+1 ±2	+11<br>–3	+1 ±0.2<br>–6 ±1	+10 ±0.2<br>+3 ±1	–55<br>–35	–35 ±3<br>–35 ±3	–20 ±3<br>0 ±3	–57<br>–40	–43 ±5<br>–30 ±0.6	–14 ±5<br>–10 ±0.6	–182<br>–123	–167 ±12; –205 ±14<br>–160 ±8	–0.4 ±0.1	–15 ±12; +23 ±14<br>+37 ±8
2010	–25<br>–35	+6 ±0.3<br>+3 ±0.1	–2.5 ±0.3	–28 ±1<br>–35 ±1	+31<br>+18	+5 ±1<br>+4 ±0.8	+26 ±1<br>+14 ±0.8	–8<br>+12	+3 ±0.2<br>+19 ±2	–11 ±0.2<br>–7 ±2	+30<br>+47	+41 ±5<br>+64 ±1	–11 ±5<br>–17 ±1	+58<br>+117	+77 ±5; +71 ±5<br>+130 ±6	–2.5 ±0.3	–16 ±5; –10 ±5<br>–10 ±7
2011	–91<br>–101	–66 ±3<br>–64 ±3	–0.4 ±0.1	–25 ±3<br>–37 ±3	+16<br>+3	+4 ±0.2<br>+2 ±0.4	+12 ±0.2<br>+1 ±0.4	–87<br>–67	–58 ±4<br>–68 ±5	–29 ±4<br>+1 ±5	–65<br>–48	–61 ±7<br>–50 ±1	–4 ±7<br>+2 ±1	–297<br>–238	–251 ±18;–293 ±21<br>–260 ±13	–0.4 ±0.1	–46 ±18; –2 ±21<br>+22 ±13
2012	+35<br>+25	+29 ±1<br>+24 ±1	–0.4 ±0.1	+6 ±1<br>+1 ±1	+8<br>–5	+1 ±0.2<br>–5 ±1	+7 ±0.2<br>0 ±1	–10<br>+10	+1 ±0.1<br>+17 ±1	–11 ±0.1<br>–7 ±1	+29<br>+46	+33 ±4<br>+55 ±1	–4 ±4<br>–9 ±1	+38<br>+97	+65 ±5; +89 ±6<br>+119 ±5	–0.4 ±0.1	–27 ±5; –51 ±6<br>–22 ±5
2003–2012	–32<br>–42	–10 ±0.4<br>–8 ±0	–4.2 ±0.4	–18 ±0.9<br>–30 ±0.5	+13<br>0	+5 ±1<br>+3 ±0.6	+8 ±1<br>–3 ±0.6	–38<br>–18	–21 ±2<br>–14 ±1	–17 ±2<br>–4 ±1	–29<br>–12	–22 ±3<br>–6 ±0.1	–7 ±3<br>–6 ±0.1	–126<br>–67	–73 ±5;–81 ±6<br>–46 ±3	–4.2 ±0.4	–49 ±5;–41 ±6<br>–17 ±3

[a]In *Method Q_1*, precipitation-based water discharge was predicted directly using post-TGD basin-wide precipitation data and the pre-TGD correlation between water discharge and precipitation in 1993–2002 . In *Method Q_2*, the precipitation-based water discharge at Datong was predicted first using post-TGD sub-basin precipitation data and the pre-TGD correlations between water discharge and precipitation of the sub-basins, and then using water delivery models for the middle and lower reaches of the main river. *Method S_2*, was omitted for the prediction of precipitation-based change in water discharge from 1950–1968 to 2003–2012, considering that the result of this method is comparable to the result of *Method S_1* as suggest by the predicted changes from 1993–2002 to 2003–2012. [b]Change measured over the post-TGD period relative to the pre-TGD averages in 1993–2002 (first row) and t 1950–2002 (second row). [c]Precipitation-based change. The error of this change is determined by Equations 22 and 23. [d]Change due to TGD operation; i.e., the loss of water discharge because of water impoundment and evaporation in the TGR. [e]Balance between the observed change and the predicted changes due to precipitation and TGD operation, presumably caused by drivers such as water consumption, other reservoirs, water-soil conservation and temperature change. The error of this predicted change for the Yichang and Datong gauging stations is determined by the errors of the predicted changes due to precipitation and TGD, i.e., being lower than the sum the errors of the predicted changes due to precipitation and TGD and larger than both of them. .

Table S3 Predicted contributions from various driving factors to post-TGD sediment-load changes (relative to pre-TGD averages in 1993–2002 (first row) and 1950–1968 (second row))

	Upstream basin at Yichang (378 Mt/y in 1993–2002; 541 Mt/yr in 1950–1968) (*Method* S_1)[a]				Hanjiang River basin (7.8 Mt/yr in 1993–2002; 119Mt/yr in 1950–1968) (*Method* S_1)[a]			Lake Dongting basin (19.3 Mt/yr in 1993–2002; 35 Mt/yr in 1950–1968) (*Method* S_1)[a]			Lake Poyang basin (10 Mt/yr in 1993–2002; 17 Mt/yr in 1950–1968) (*Method* S_1)[a]			Basin upstream from Datong (tidal limit) (320 Mt/yr in 1993–2002; 507 Mt/yr in1950–1968 (*Method* S_1; *Method* S_2) [a]			
	Observed changes (Mt/yr)[b]	Predicted changes (Mt/yr)[c]	Predicted changes (Mt/yr) [d]	Predicted changes (Mt/yr)[e]	Observed changes (Mt/yr)[b]	Predicted changes (Mt/yr)[c]	Predicted changes (Mt/yr)[e]	Observed changes (Mt/yr)[b]	Predicted changes (Mt/yr)[c]	Predicted changes (Mt/yr)[e]	Observed changes (Mt/yr)[b]	Predicted changes (Mt/yr)[c]	Predicted changes (Mt/yr)[e]	Observed changes (Mt/yr)[b]	Predicted Changes (Mt/yr)[c]	Predicted changes (Mt/yr) [d]	Predicted Changes (Mt/yr)[e]
2003	–280<br>–443	–9 ±2<br>–20 ±3	–166 ±6	–43 ±7<br>–257 ±8	+7.4<br>–104	+7.9 ±3<br>+106 ±41	–0.4 ±3<br>–210 ±41	–1.9<br>–17	–8.6 ±3<br>–5.9 ±2	+6.7 ±3<br>–11 ±2	–4.7<br>–12	–5.1 ±2<br>–6.2 ±1	+0.4 ±2<br>–5.5 ±1	–114<br>–301	–24 ±4; –27 ±5<br>–15 ±2	–113 ±7	+23 ±9; +26 ±9<br>–173 ±8
2004	–314<br>–477	+66 ±15<br>+24 ±3	–149 ±4	–230 ±17<br>–352 ±6	–0.2<br>–111	–1.3 ±0.5<br>–17 ±8	+1.1 ±0.5<br>–94 ±8	–10<br>–26	–3.9 ±1<br>-0.4 ±0.2	–6.1 ±1<br>–26 ±0.2	–6.9<br>–14	–4.6 ±1<br>–5.2 ±1	–2.3 ±1<br>–8.8 ±1	–173<br>–360	–13 ±2; +6 ±1<br>+12 ±2	–87 ±5	–73 ±6;–92 ±6<br>–285 ±6
2005	–268<br>–431	–18 ±4<br>–25 ±3	–195 ±5	–55 ±8<br>–211 ±7	+9.3<br>–102	+3.9 ±2<br>+58 ±22	+6.1 ±2<br>–160 ±22	–13<br>–29	–6.5 ±2<br>–3.3 ±1	–6.5 ±2<br>–26 ±1	–3.1<br>–10	–2.2 ±0.6<br>+0.7 ±0.2	–0.9 ±0.6<br>–11 ±0.2	–104<br>–291	–24 ±4; –18 ±3<br>–15 ±2	–110 ±6	+30 ±8; +24 ±8<br>–166 ±7
2006	–369<br>–532	–145 ±32<br>–116 ±15	–116 ±2	–107 ±31<br>–300 ±16	–5.0<br>–120	–3.8 ±1<br>–4.3 ±2	–1.2 ±1<br>–112 ±2	–9.0<br>–25	–5.3 ±2<br>–2 ±0.8	–3.7 ±2<br>–23 ±0.4	–2.8<br>–9.8	–1.6 ±0.4<br>+2.1 ±0.4	–1.2 ±0.4<br>–12 ±0.4	–235<br>–322	–44 ±7; –69 ±10<br>–64 ±9	–77 ±3	–114 ±9;–89 ±12<br>–181 ±11
2007	–325<br>–488	–19 ±4<br>–26 ±3	–202 ±5	–104 ±8<br>–260 ±7	+0.5<br>–111	–0.3 ±0.1<br>+1.2 ±0.4	+0.8 ±0.1<br>–112 ±0.4	–11<br>–27	–8.8 ±3<br>–6.1 ±2	–2.2 ±3<br>–21 ±2	–7.1<br>–14	–5.4 ±±2<br>–6.9 ±1	–1.7 ±2<br>–7.1 ±1	–182<br>–369	–38 ±6; –37 ±6<br>–50 ±8	–105 ±6	–37 ±9;–40 ±9<br>–214 ±12
2008	–346<br>–509	+37 ±8<br>+7 ±0.9	–235 ±6	–148 ±12<br>–280 ±7	–3.2<br>–114	–0.2 ±0.1<br>+0.8 ±0.3	–3.1 ±0.1<br>–115 ±0.3	–11<br>–27	–3.8 ±1<br>-0.4 ±0.2	–7.2 ±1<br>–27 ±0.2	–8.1<br>–13	–5.5 ±1<br>–2.7 ±0.6	–2.6 ±1<br>–10 ±0.6	–190<br>–377	–8 ±1; +1 ±0.2<br>+22 ±3	–136 ±7	–46 ±8; –55 ±7<br>–263 ±9
2009	–343<br>–506	–106 ±23<br>–85 ±11	–175 ±4	–62 ±25<br>–246 ±13	–2.9<br>–114	–2.5 ±1<br>–1.9 ±2	–0.4 ±1<br>–112 ±2	–16<br>–32	–9.4 ±3<br>–6.9 ±3	–6.6 ±3<br>–25 ±3	–7.0<br>–14	–4.9 ±2<br>–5.1 ±1	–2.1 ±2<br>–8.9 ±1	–209<br>–396	–54 ±8; –71 ±10<br>–87 ±13	–108 ±5	–47 ±11; –30 ±13<br>–201 ±16
2010	–345<br>–508	+2 ±0.4<br>–13 ±2	–224 ±4	–119 ±4<br>–271 ±5	+4.6<br>–107	0 ±0<br>+4.2 ±0.8	+4.6 ±0<br>–111 ±0.8	–6.8<br>–23	–0.9 ±0.3<br>+2.5 ±1	–5.9 ±0.3<br>–26 ±1	+2.8<br>–4.2	+4.6 ±2<br>+17 ±4	–1.8 ±2<br>–21 ±4	–135<br>–322	+22 ±3; +16 ±2<br>+95 ±14	–151 ±7	–6 ±7; 0 ±3<br>–266 ±18
2011	–372<br>–535	–133 ±29<br>–106 ±14	–114 ±4	–125 ±31<br>–315 ±16	–2.4<br>–114	–0.6 ±0.2<br>–5.6 ±2	–1.8 ±0.2<br>–99 ±2	–17<br>–33	–13 ±5<br>–12 ±4	–4 ±3<br>–22 ±4	–6.0<br>–13	–6.4 ±2<br>–9.5 ±2	+0.4 ±2<br>–3.5 ±2	–248<br>–485	–80 ±12; –98 ±15<br>–150 ±23	–78 ±5	–90 ±15; –72 ±18<br>–257 ±26
2012	–335<br>–498	+53 ±12<br>+16 ±2	–217 ±6	–171 ±15<br>–297 ±7	–4.1<br>–115	–2.4 ±1<br>–1.7 ±0.7	–1.7 ±1<br>–113 ±0.7	–13<br>–29	–1.3 ±0.5<br>+2.1 ±1	–12 ±0.5<br>–31 ±1	–1.0<br>–8.0	+3.5 ±1<br>+15 ±4	–4.5 ±1<br>–23 ±4	–159<br>–346	+18 ±3; +28 ±4<br>+87 ±13	–157 ±7	–20 ±9; –30 ±9<br>–276 ±17
Average in 2003–2012	–330<br>–493	–27 ±6<br>–35 ±4	–179 ±5	–114 ±9<br>–279 ±7	+0.5<br>–111	+0.1 ±0<br>+1.6 ±0.6	+0.4 ±0<br>–113 ±0.6	–11<br>–27	–6.1 ±2<br>–3.3 ±1	–4.9 ±2<br>–24 ±1	–4.2<br>–11	–2.5 ±0.7<br>-0.1 ±0	–1.7 ±0.7<br>–11 ±0	–175<br>–362	–24 ±4; –27 ±5<br>–17 ±3	–113 ±6	–39 ±8; –36 ±9<br>–233 ±8

[a] In *Method* S_1, the precipitation-based sediment discharge was predicted directly using post-TGD basin-wide precipitation data and the pre-TGD correlations between precipitation and water and sediment discharge. In *Method* S_2, the precipitation-based sediment discharge at Datong was predicted firstly using the post-TGD sub-basin precipitation data and the pre-TGD correlations between precipitation and water and discharge of the sub-basins, and then using sediment delivery models for the middle and lower reaches of the main river. *Method* S_2, was omitted for the prediction of precipitation-based change in sediment flux from 1950–1968 to 2003–2012, considering that the result of this method is comparable to the result of *Method* S_1 as suggest by the predicted changes from 1993–2002 to 2003–2012. [b]Change measured over the post-TGD period relative to the pre-TGD averages in 1993–2002 (first row) and 1956–2002 (second row). [c]Change generated by precipitation; i.e., the difference between the post-TGD precipitation-based sediment discharge and the average pre-TGD sediment discharge. The error of this change is determined by Equations 22 and 23. [d]Change in sediment discharge due to the TGD operation; i.e., the impact of sedimentation in the TGR and downstream deposition/erosion in response to TGD operation. For the Yichang station, the error of predicted change in sediment load is basically the error in estimating the sediment flux from the ungauged areas around the TGR, assuming that the error of sediment discharge at the gauging stations is negligible. The annual sediment fluxes from the ungauged areas in 2003–2012 were predicted by Yang et al. (2014) (see their Table). In this study, we estimated the relative errors of their predictions using Equation 22 and 23. [e]Balance between observed and predicted change due to precipitation and TGD operation, presumably caused by drivers such as other dams, water-soil conservation, sand mining, water consumption, and temperature change. The error of this predicted change is determined by the errors of the predicted changes due to precipitation and TGD.

Table S4 An example of error estimation for predicted water discharges at Datong using the regression relationship between annual precipitation and water discharge

	Measured values		Predicted *Q* values ((km^3/yr)		Errors of predicted *Q* values (km^3/yr)				Relative errors of predicted *Q* values (%)			
	P (mm/yr)	*Q* (km^3/yr)	Regression of odd years[1]	Regression of even years[2]	Odd-year regression for even years	Odd-year regression for odd years	Even-year regression for even years	Even-year regression for odd years	Odd-year regression for even years	Odd-year regression for odd years	Even-year regression for even years	Even-year regression for odd years
1956	1028	843	873	883	30		40		4		5	
1957	1022	824	864	874		40		50		5		6
1958	1005	862	841	850	-21		-12		-2		-1	
1959	1016	773	856	866		83		93		11		12
1960	973	768	798	807	30		39		4		5	
1961	1058	892	913	924		21		32		2		4
1962	1031	941	876	886	-65		-55		-7		-6	
1963	979	770	807	816		37		46		5		6
1964	1065	1038	922	933	-116		-105		-11		-10	
1965	1063	875	919	930		44		55		5		6
1966	904	775	704	712	-71		-63		-9		-8	
1967	1063	888	920	930		32		42		4		5
1968	1086	944	950	962	6		18		1		2	
1969	1027	874	871	881		-3		7		0		1
1970	1119	992	996	1008	4		16		0		2	
1971	927	731	736	744		5		13		1		2
1972	956	698	774	783	76		85		11		12	
1973	1145	1074	1031	1043		-43		-31		-4		-3
1974	1040	839	888	899	49		60		6		7	
1975	1126	1008	1004	1016		-4		8		0		1
1976	975	842	800	809	-42		-33		-5		-4	
1977	1025	914	869	878		-45		-36		-5		-4
1978	922	676	729	737	53		61		8		9	
1979	950	738	767	775		29		37		4		5
1980	1146	997	1032	1044	35		47		4		5	
1981	1017	878	857	866		-21		-12		-2		-1
1982	1099	957	968	979	11		22		1		2	
1983	1197	1110	1101	1114		-9		4		-1		0
1984	1030	874	874	884	0		10		0		1	
1985	991	823	822	832		-1		9		0		1
1986	916	714	721	728	7		14		1		2	
1987	1033	837	879	889		42		52		5		6
1988	940	832	753	762	-79		-70		-9		-8	
1989	1103	966	973	985		7		19		1		2
1990	1042	906	891	901	-15		-5		-2		-1	
1991	1057	929	911	921		-18		-8		-2		-1
1992	993	869	824	834	-45		-35		-5		-4	
1993	1147	977	1033	1045		56		68		6		7
1994	1048	853	900	910	47		57		6		7	
1995	1033	980	879	889		-101		-91		-10		-9
1996	1017	952	857	867	-95		-85		-10		-9	
1997	980	850	807	816		-43		-34		-5		-4
1998	1211	1244	1120	1134	-124		-110		-10		-9	
1999	1116	1037	992	1004		-45		-33		-4		-3
2000	1086	927	950	961	23		34		2		4	
2001	949	825	765	774		-60		-51		-7		-6
2002	1158	993	1048	1061	55		68		6		7	
A ±S					–10 ±57	0 ±43	0 ±57	10 ±43	–1 ±6	0 ±5	0 ±6	1 ±5

[1] $Q = 1.3513\ P – 517$, $r = 0.91$, $p < 0.001$. [2] $Q = 1.3727\ P – 529$, $r = 0.89$, $p < 0.001$. Error of predicted *Q*: The difference between predicted and measured water discharge. Relative error of predicted *Q*(%): (Predicted *Q* —Measured *Q*) *100/ Measured *Q*. A: average; S: Standard deviation.

Table S5 Errors of predicted water and sediment discharges at Yichang and Datong stations for the pre-TGD decade (1993–2002), using the regression relationships between annual precipitation and water discharge (Equation 2 and 6) and between water discharge and sediment flux (Equations 11 and 15) over the pre-TGD decade

	For the gauging station at Yichang							For the gauging station at Datong (Tidal limit)						
	Measured values			Predicted values±S		Relative errors		Measured values			Predicted values±S		Relative errors	
	P (mm/yr)	Q (km^3/yr)	Q_S (Mt/yr)	Q (km^3/yr)	Q_S (Mt/yr)	Q (%)	Q_S (%)	P (mm/yr)	Q (km^3/yr)	Q_S (Mt/yr)	Q (km^3/yr)	Q_S (Mt/yr)	Q (%)	Q_S (%)
1993	917	460	464	477±20	471±106	4	1	1147	977	370	1049±74	344±46	7	–7
1994	789	348	210	379±15	269±60	9	28	1048	853	239	932±63	308±41	9	29
1995	855	423	363	429±17	365±82	2	1	1033	980	355	914±64	302±41	–7	–15
1996	813	422	359	398±16	302±68	–6	–16	1017	952	324	894±63	296±41	–6	–9
1997	753	363	337	352±14	225±50	–3	–33	980	850	299	850±59	282±40	0	–6
1998	968	523	743	515±21	570±127	–2	–23	1211	1244	401	1126±79	368±48	–9	–8
1999	916	482	433	476±19	469±105	–1	8	1116	1037	317	1013±71	333±49	–2	5
2000	906	471	390	468±19	451±101	–1	16	1086	927	339	977±69	322±44	5	–5
2001	813	416	299	397±16	301±67	–4	1	949	825	276	814±58	271±43	–1	–2
2002	829	393	228	410±17	326±73	4	43	1158	993	275	1063±74	348±36	7	27
Average in 1993–2002	856	430	383	430±18	375±84	0±5	3±23	1075	964	320	963±68	317±42	0 ±7	–1 ±15

P: Precipitation; Q: Water discharge; Q_S: Sediment flux; S: Prediction error estimated by Equations 22 and 23.. Relative error: $(P_v - M_V)$*100/M_V, where P_v and M_V represent predicted and measured values, respectively.
